# New Ferulic Acid and Amino Acid Derivatives with Increased Cosmeceutical and Pharmaceutical Potential

**DOI:** 10.3390/pharmaceutics15010117

**Published:** 2022-12-29

**Authors:** Ewa Janus, Luan Ramalho Pinheiro, Anna Nowak, Edyta Kucharska, Ewelina Świątek, Natalia Podolak, Magdalena Perużyńska, Katarzyna Piotrowska, Wiktoria Duchnik, Łukasz Kucharski, Adam Klimowicz

**Affiliations:** 1 Department of Chemical Organic Technology and Polymeric Materials, Faculty of Chemical Technology and Engineering, West Pomeranian University of Technology in Szczecin, Pulaski Ave. 10, 70-322 Szczecin, Poland; 2 Polytechnic Institute of PUC Minas, Pontifical Catholic University of Minas Gerais, Belo Horizonte 30535-901, Brazil; 3 Department of Cosmetic and Pharmaceutical Chemistry, Pomeranian Medical University in Szczecin, 70-111 Szczecin, Poland; 4 Department of Experimental & Clinical Pharmacology, Pomeranian Medical University in Szczecin, 70-111 Szczecin, Poland; 5 Department of Physiology, Pomeranian Medical University in Szczecin, 70-111 Szczecin, Poland

**Keywords:** antioxidant activity, antiaging, ferulic acid, new ferulic acid and amino acid derivatives, skin permeation, toxicity, vehicles containing new ferulic acid and amino acid derivatives

## Abstract

Ferulic acid (FA) has been widely used in the pharmaceutical and cosmetics industry due to its, inter alia, antioxidant, antiaging and anti-inflammatory effects This compound added to cosmetic preparations can protect skin because of its photoprotective activity. However, the usefulness of FA as a therapeutic agent is limited due to its low solubility and bioavailability. The paper presents the synthesis, identification, and physicochemical properties of new FA derivatives with propyl esters of three amino acids, glycine (GPr[FA]), L-leucine (LPr[FA]), and L-proline (PPr[FA]). The NMR and FTIR spectroscopy, DSC, and TG analysis were used as analytical methods. Moreover, water solubility of the new conjugates was compared with the parent acid. Both ferulic acid and its conjugates were introduced into hydrogel and emulsion, and the resulting formulations were evaluated for stability. Additionally, in vitro penetration of all studied compounds from both formulations and for comparative purposes using Franz diffusion cells was evaluated from the solution in 70% (*v*/*v*) ethanol. Finally, cytotoxicity against murine fibroblasts L929 was tested. All of the analyzed compounds permeated pig skin and accumulated in it. LPr[FA] and PPr[FA] were characterized by much better permeability compared to the parent ferulic acid. Additionally, it was shown that all the analyzed derivatives are characterized by high antioxidant activity and lack of cytotoxicity. Therefore, they can be considered as an interesting alternative to be applied in dermatologic and cosmetic preparations.

## 1. Introduction

Ferulic acid (FA) belongs to the group of polyphenols and has been widely used in the pharmaceutical and cosmetics industries. This compound naturally occurs in plants, especially in the Umbrella family Ranunculaceae and Gramineae. It occurs frequently in grains, spinach, parsley, grapes, rhubarb, and cereal seeds, mainly wheat, oats, rye, and barley [1]. In plants, it is often cross-linked with lignin and polysaccharide to form part of the plant cell wall [2]. FA is characterized primarily by antioxidant activity [1,3,4], by which it provides skin protection against UV-induced erythema. Exogenous antioxidant substances play a very important role in skin protection. According to Maya-Cano et al., the skin is exposed to reactive oxygen species (ROS). ROS are frequently formed as secondary products of some metabolic pathways and also by specific systems, such as the pathological inflammatory processes, oxidases, cytokines, peroxisomes, xanthine oxidase (XO), NADPH oxidase, acetyl CoA oxidase, or cytochromes [5]. A key role in oxidative stress is played by superoxide anions (O_2_^−^), hydroxyl, nitric oxide radicals, and hydrogen peroxide (H_2_O_2_). One of the main factors responsible for the harmful effect of ROS on the skin is long-term skin exposure to ultraviolet (UV) radiation [5,6]. Other factors include environmental pollution, smoking, and an incorrect diet. All these factors lead to faster skin aging, inflammatory processes, or even skin cancer [7]. Due to its valuable properties, an increased interest in using FA in cosmetics and dermatological products has been seen in recent years. Its properties are appreciated in photo-protective, antiaging and brightening cosmetic preparations. In addition, it inhibits melanogenesis, enhances angiogenesis, and accelerates wound healing. FA may also inhibit MMP-1 and MMP-9 expression in human dermal fibroblast lines as well as induce procollagen synthesis. It reduces MMP-1 production after UVB irradiation and can also increase cell viability after UVB irradiation of the fibroblasts. Tsay et al. demonstrated a healing effect on fibroblasts, with the percentage of viable cells increased from 9.92% to 46.02% [8]. FA exerts beneficial effects on major skin structures, including keratinocytes, fibroblasts, collagen, and elastin [1]; moreover, it is characterized by low toxicity [9]. Despite many beneficial properties of ferulic acid, its usefulness as a therapeutic agent is limited due to its low solubility and bioavailability [10]. However, its low permeation into/across the skin may be insufficient to exert a pharmacological effect [1]. The absorption and penetration of compounds through the skin depends on their physicochemical properties, in particular lipophilicity [11,12]. The main barrier limiting the penetration of active substances through the skin is the stratum corneum (SC), which prevents excessive water loss, as well as the penetration of microorganisms, allergens, and chemicals [13,14]. Its main components are lipids, such as cholesterol and its esters, ceramides, and fatty acids [15]. Therefore, the active compounds are constantly being modified to increase their skin permeation [16,17,18]. Due to the low FA bioavailability, its derivatives could be taken into account as alternatives. FA contains phenyl, hydroxyl, and carboxyl groups, an ethylenic bond, and a benzene ring. Therefore, the structural characteristics of FA make it an optimal substrate to synthesize various derivatives [19] with increased skin penetration. For example, such derivatives as ferulic acid ethyl ether (FAEE), coniferyl aldehyde (CD), and coniferyl alcohol (CA) also permeated the skin to a higher degree than pure FA. However, Zhang et al. did not observe a difference in permeation using two different buffers in the donor chamber (pH 6 and 9.9) [17]. Several previous studies reported on FA derivatives that may be of potential use in cosmetology and pharmacology. FA derivatives such as the ferulic acid ethyl ester, (FAEE), protected skin melanocytes against UV-induced oxidative stress and cell damage. Treatment with FAEE reduced the production of ROS in UVB irradiated human melanocytes [20]. The antioxidant effect of FA is of importance in the probable treatment of other diseases. For example, treatment with FA of diabetic rats (50 mg kg^−1^ body wt., orally for 8 weeks) markedly ameliorated kidney injury, renal cell apoptosis, inflammation, and defective autophagy in the kidneys. In cultured NRK-52E cells, ferulic acid (at an optimum dose of 75 μM) countered excessive ROS generation, induced autophagy, and inhibited apoptotic death of cells under high glucose environment [21].

Poor water solubility of ferulic acid limits its use and the possibility of incorporating its high concentrations into the hydrophilic topical formulations. Therefore, different methods of solubility enhancement are sought. An interesting approach is to modify poorly soluble, biologically active acids by forming their salts. The selection of the cation is important here, and recently, organic cations have gained several advantages, in particular biocompatible cations such as choline. Ferulic acid and other hydroxycinnamic acids have been converted to the cholinium salts which demonstrated a higher water solubility and antioxidant properties in comparison to their acidic precursors. In addition, these derivatives were characterized by negligible cytotoxicity activity [22,23]. Other authors disclosed an approach where they used cholinium salts of amino acids as green excipients to enhance the solubility and content of ferulic acid in formulation. This allowed for higher activity of the preparations [24]. In turn, our research on the modification of non-steroidal anti-inflammatory drugs with alkyl esters of various amino acids showed a significant effect of both the amino acid part and the alkyl group in the ester part on the solubility of the active substance and its permeability through the skin [16,25,26]. Taking into account the above data, the aim of the presented studies was to modify ferulic acid with propyl esters of three amino acids—glycine, L-leucine, L-proline—which are essential for maintaining the proper structure of the skin and to determine the solubility of the obtained derivatives and the possibility of their use in cosmetics preparations.

## 2. Materials and Methods

### 2.1. Materials

Ferulic acid (98%) was supplied by AmBeed (Arlington Hts, IL, USA). Glycine (G), L-leucine (L) and L-proline (P) (≥99%) were provided by Carl Roth (Karlsruhe, Germany). Trimethylsilyl chloride (≥99%) (TMSCl) was purchased from Sigma-Aldrich (Steinheim am Albuch, Germany). Ammonium hydroxide solution 25% (NH_3_·H_2_O) of analytical grade was purchased from StanLab (Lublin, Poland). Propan-1-ol (PrOH), chloroform, diethyl ether of high purity were supplied by Chempur (Piekary Śląskie, Poland). Deuterated chloroform (CDCl_3_) (99.8%) (+0.03% TMSCl) was purchased from Eurisotop (Cheshire, UK). Deuterated dimethyl sulfoxide (DMSO-d_6_) (99.8%) was from Deutero GmbH (Kastellaun, Germany). 2,2-diphenyl-1-picrylhydrazyl (DPPH), 6-hydroxy-2,5,7,8-tetramethylchroman-2-carboxylic acid (Trolox), glucose medium, L-glutamine, penicillin/streptomycin were purchased from Sigma Aldrich (Sigma-Aldrich Merck Group, St. Louis, MO, USA); bovine serum was purchased from EURx (Gdansk, Poland). Folin–Ciocalteu reagent and gallic acid were from Merck, (Darmstadt, Germany); 99.5% acetic acid, 96% ethanol, methanol, glycerin, and phosphate-buffered saline (PBS; pH 7.00 ± 0.05) were from Chempur (Piekary Śląskie, Poland), whereas acetonitrile (J.T. Baker, Radnor, PA, USA) for HPLC was supplied by Avantor Performance Materials Poland S.A. (Gliwice, Poland). Biobase, hydroxyethylcellulose, and beeswax were purchased from Mazidła.com (Poznań, Poland); grape seed oil was purchased from Monini (Spoleto, Italy). All reagents were of analytical grade.

### 2.2. Synthesis of the Amino Acid Propyl Ester Ferulates AAPr[FA] 

**Scheme 1:**
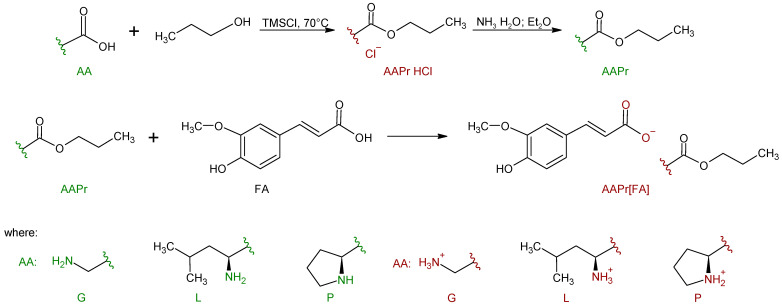
The modification path of ferulic acid with amino acid propyl esters.

Synthesis of the ferulic acid derivatives consisted of three steps (Scheme 1). In the first step, an amino acid (glycine, L-leucine, L-proline) was esterified with propyl alcohol to obtain the proper amino acid propyl ester hydrochloride (AAPr HCl). In the second step, the prepared ester hydrochloride was neutralized to the amino acid propyl ester (AAPr). Esterification and neutralization reactions were conducted by the modified, earlier described method [16]. The third synthesis step was the combination of ferulic acid with the prepared AAPr. These three synthesis steps were conducted as described below. 

Briefly, 0.1 Mol of amino acid (AA) was suspended in propyl alcohol used in 10-fold molar excess and then the apparatus was flushed with argon. While vigorously stirring the suspension at room temperature, 3 molar equivalents of trimethylsilyl chloride (TMSCl) were added dropwise. Then the mixture was heated to the temperature of 70 °C and maintained under these conditions for 24 h or until the suspension disappeared. The excess of TMSCl, propyl alcohol, and formed by-products were removed by evaporation at 60 °C under reduced pressure. The obtained crude product was purified by washing with diethyl ether. Pre-purified product was next dissolved in chloroform to remove possible residues of unreacted amino acid. The solution was then filtered when needed and chloroform was distilled off from the filtrate on a rotavapor under reduced pressure. Then the purified product was dried in a vacuum dryer at 60 °C for 24 h. As a result, the amino acid propyl ester hydrochloride, AAPr HCl was obtained with a high yield 95–97%. The identification and purity evaluation of AAPr HCl was performed based on the NMR spectroscopy.

Then, the synthesized AAPr HCl was dissolved in small amount of distilled water and neutralized by the addition of five molar equivalents of 25% ammonium hydroxide aqueous solution. The solution was intensively mixed and then, the product was extracted three times with diethyl ether. The organic layers were collected and dried using anhydrous Na_2_SO_4_. Then, diethyl ether was distilled off to receive amino acid propyl ester (AAPr) with the yield in the range of 55–80%. Immediately after preparation, the respective AAPr was used to modify ferulic acid. For this purpose, the equimolar amounts of the formed AAPr and ferulic acid were weighed and placed in a mortar with a small amount of ethanol (0.5–1 mL). The reactants were thoroughly ground in a mortar to a homogeneous form. Then the product was placed in a vacuum dryer at 50 °C and under pressure of 5 mbar for 8 h to remove moisture and ethanol residues. The derivatives of ferulic acid and amino acid propyl esters were obtained as white solids.

GPr[FA]—glycine propyl ester ferulate.

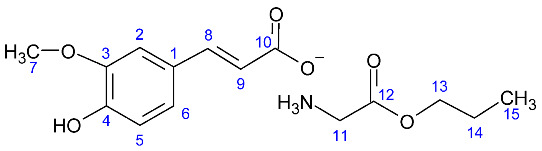



Yield: 97%; ^1^H NMR (400 MHz, DMSO-d_6_) δ in ppm: 7.44 (d, J_8,9_ = 15.9 Hz, 1H_8_), 7.25 (d, J_2,6_ = 1.8 Hz, 1H_2_), 7.05 (dd, J_6,5_ = 8.2, J_6,2_ = 1.8 Hz, 1H_6_), 6.78 (d, J_5,6_ = 8.1 Hz, 1H_5_), 6.36 (d, J_9,8_ = 15.9 Hz, 1H_9_), 4.00 (t, J_13,14_ = 6.7 Hz, 2H_13_), 3.80 (s, 3H_7_), 3.32 (s, 2H_11_), 1.58 (m, J_14,15_ = 7.3 Hz, 2H_14_), 0.88 (t, J_15,14_ = 7.4 Hz, 3H_15_). ^13^C NMR (100 MHz, DMSO-d_6_) δ in ppm: 173.61 (C_12_), 168.37 (C_10_), 148.98 (C_3_), 147.92 (C_4_), 143.74 (C_8_), 125.94 (C_1_), 122.65 (C_6_), 116.66 (C_9_), 115.52 (C_5_), 111.02 (C_2_), 65.54 (C_13_), 55.67 (C_7_), 43.06 (C_11_), 21.59 (C_14_), 10.28 (C_15_). FT-IR (ATR) ν cm^−1^: 517, 572, 699, 731, 815, 868, 909, 931, 981, 992, 1036, 1059, 1118, 1171, 1228, 1275, 1378, 1427, 1452, 1508, 1558, 1587, 1602, 1637, 1745, 2880, 2968, 3215.

LPr[FA]—L-leucine propyl ester ferulate.

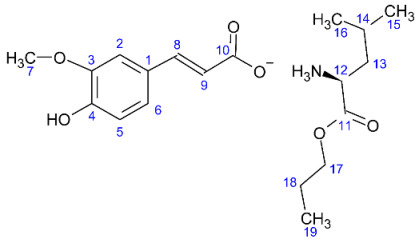



Yield: 98%; ^1^H NMR (400 MHz, DMSO-d_6_) δ in ppm: 7.46 (d, J_8,9_ = 15.9 Hz, 1H_8_), 7.26 (d, J_2,6_ = 1.8 Hz, 1H_2_), 7.06 (dd, J_6,5_ = 8.2, J_6,2_ = 1.8 Hz, 1H_6_), 6.78 (d, J_5,6_ = 8.1 Hz, 1H_5_), 6.36 (d, J_9,8_ = 15.9 Hz, 1H_9_), 3.99 (m, 2H_17_), 3.81 (s, 3H_7_), 3.33 (dd, 1H_12_), 1.71 (m, 1H_13_), 1.58 (m, 2H_18_), 1.44–1.29 (m, 1H_13_, 1H_14_), 0.84–0.90 (m, 3H_15,_ 3H_1__6,_ 3H_19_). ^13^C NMR (100 MHz, DMSO-d_6_) δ in ppm: 175.98 (C_11_), 168.22 (C_10_), 149.02 (C_3_), 147.91 (C_4_), 144.02 (C_8_), 125.87 (C_1_), 122.72 (C_6_), 116.27 (C_9_), 115.51 (C_5_), 111.05 (C_2_), 65.45 (C_17_), 55.66 (C_7_), 52.34 (C_12_), 43.66 (C_14_), 24.20(C_16_), 22.78 (C_15_), 21.95 (C_13_), 21.58 (C_18_), 10.30 (C_19_). FT-IR (ATR) ν cm^−1^: 453, 508, 525, 571, 588, 604, 713, 744, 815, 863, 926, 958, 978, 1036, 1060, 1089, 1126, 1160, 1195, 1209, 1267, 1304, 1368, 1404, 1454, 1467, 1517, 1591, 1645, 1736, 2834, 2865, 2934, 2959, 3203.

PPr[FA]—L-proline propyl ester ferulate.

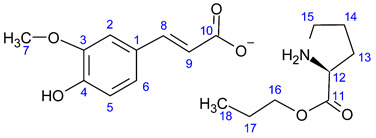



Yield: 94%; ^1^H NMR (400 MHz, DMSO-d_6_) δ in ppm: 7.46 (d, J_8,9_ = 15.9 Hz, 1H_8_), 7.26 (d, J_2,6_ = 1.8 Hz, 1H_2_), 7.06 (dd, J_6,5_ = 8.2, J_6,2_ = 1.8 Hz, 1H_6_), 6.78 (d, J_5,6_ = 8.1 Hz, 1H_5_), 6.36 (d, J_9,8_ = 15.9 Hz, 1H_9_), 3.99 (m, 2H_16_), 3.81 (s, 3H_7_), 3.68 (dd, 1H_12_), 2.90 (m, 1H_15_), 2.77 (m, 1H_15_), 1.98 (m, 1H_13_), 1.74–1.54 (m, 1H_13_, 2H_14_, 2H_17_), 0.88 (t, 3H_18_). ^13^C NMR (100 MHz, DMSO-d_6_) δ in ppm: 174.60 (C_11_), 168.23 (C_10_), 149.01 (C_3_), 147.91 (C_4_), 144.06 (C_8_), 125.86 (C_1_), 122.73 (C_6_), 116.22 (C_9_), 115.51 (C_5_), 111.05 (C_2_), 65.61 (C_16_), 59.07 (C_12_), 55.67 (C_7_), 46.34 (C_15_), 29.64 (C_13_), 25.12 (C_14_), 21.57 (C_17_), 10.26 (C_18_). FT-IR (ATR) ν cm^−1^: 455, 494, 529, 570, 581, 604, 697, 744, 778, 806, 825, 858, 885, 897, 931, 965, 986, 1022, 1055, 1121, 1159, 1177, 1215, 1275, 1373, 1422, 1453, 1518, 1585, 1602, 1635, 1739, 2966.

### 2.3. Methods Used for Identification and Characterization of Ferulic Acid Derivatives 

The NMR spectra for ferulic acid and its derivatives were recorded in CDCl_3_ or in DMSO-d_6_ on a BRUKER DPX-400 spectrometer (Billerica, MA, USA) operating at 400.13 MHz (^1^H) and 100.62 MHz (^13^C). CDCl_3_ as solvent and TMSCl as chemical shift standard were used for amino acid propyl ester hydrochlorides and DMSO-d_6_ for ferulic acid and its derivatives. 

The FTIR spectra were recorded using a spectrometer model ‘Nicolet 380′ (Thermo Electron Corporation, Waltham, MA, USA) with Diamond ATR in transmission mode, in the range of 4000–400 cm^−^^1^. The resolution was 4 cm^−^^1^.

Thermogravimetric (TG) analysis was carried out using thermomicrobalance TG 209 F1 Libra^®^ from NETZSCH (Selb, Germany). Samples of 2–5 mg, loaded in aluminium crucibles, were heated from 30 °C to 600 °C at the heating rate of 10 °C·min^−1^, in the air atmosphere (25 mL·min^−1^) with nitrogen flow (10 mL·min^−1^) as the purge gas. 

Differential scanning calorimetry (DSC) was performed using TA Instruments, model DSC 250 (New Castle, DE, USA). The sample was loaded on an aluminum pan with a lid. The analysis was carried out in nitrogen atmosphere. The sample was first heated from 20 °C to specified temperature (210 °C for ferulic acid, 170 °C for GPr[FA], 140 °C for LPr[FA] and 130 °C for PPr[FA]), then cooled to 20 °C and again heated to the specified temperature. The rate of heating/cooling/heating was 10 °C·min^−^^1^. Indium and mercury were used as standards to calibrate the temperature. 

The specific rotation [α]_D_^20^ was measured in ethanol solution using AUTOPOL IV Polarimeter (Rudolph Research Analytical, Hackettstown, NJ, USA) for the concentration of compound of 0.01 g·cm^−^^3^. A polarimeter precision was 0.001° of angular rotation, and accuracy of temperature determination was 0.1 °C.

The Agilent 1200 Series HPLC system of Agilent Technologies Inc. (Santa Clara, USA), consisting of Quaternary pump, Vacuum degasser, Manual Injector, Diode Array Detector and Thermostatted Column Compartment equipped with Kinetex^®^F5 column (2.6 µm; 150 mm × 4.6 mm; Phenomenex, Torrance, CA, USA) was used in solubility experiments. Analyses were performed at 30 °C under isocratic conditions, with mobile phase consisting of water/acetonitrile mixture (50/50, *v*/*v*) and a flow rate of 1 mL min^−^^1^. Detection wavelength was 215 nm. Instrument control and data acquisition and processing were performed using an Agilent ChemStation software. Injections were repeated at least three times for each sample and the results were averaged. Concentration of ferulic acid and its salts were calculated based on peak area measurements using a calibration curve method.

Solubility in deionized water was determined for ferulic acid and its salts with amino acid propyl esters and defined as saturation concentration—a maximum quantity of a substance that may be dissolved at a given temperature per volume unit of solvent. An excess of the compound was added to 2 mL of water in screwed vials and was stirred vigorously at 25.00 ± 0.05 °C for 24 h. Then mixture was centrifuged and liquid above the solid was taken, diluted, and analyzed by the HPLC method to determine the concentration of the compound. Three independent solubility test replicates were performed for each compound.

The *n*-octanol/water partition coefficient (log P_O/W_) was determined for ferulic acid and its derivatives by shake flask method. *n*-Octanol saturated with water and water saturated with *n*-octanol were prepared at 25 °C, 24 h prior to their use in the experiments. Then, a sample of 10 mg of compound was weighed with the accuracy of 0.01 mg in a 16 cm^3^ screwed vial and dissolved in 5 cm^3^ of *n*-octanol. Next, 5 cm^3^ of water was added to the vial. The resulting two-phase mixture was kept in water bath shaker at 25 °C for 2 h and was strongly shaken to reach the partition equilibrium of the compound between the phases. Then the mixture was left unshaken for 30 min and 1.0 cm^3^ of water phase was carefully taken out to centrifuge tube. The sample was next centrifuged for 10 min at 7500 rpm at 25 °C to ensure the most precise phase separation. The concentration of compound in water phase was then determined using HPLC method. The partition coefficient, log P_O/W_ was calculated according to the formula:

$$\log P_{O/W}=\log\left(\frac{C_{0}V_{0}-C_{W}V_{W}}{C_{W}V_{W}}\right)$$

where: C_0_—initial concentration of the compound in *n*-octanol, calculated based on the weighed mass of compound, mg·cm^−^^3^; V_0_—volume of *n*-octanol, cm^3^; C_W_—concentration of the compound in water after partition equilibrium, mg·cm^−^^3^; V_W_—volume of water, cm^3^.

### 2.4. Hydrogel and Emulsion Preparation with Ferulic Acid and Its Derivatives

The hydrogel was prepared according to a modified procedure by Zagórska-Dziok et al. and Nowak et al. [6,27]. The hydroxyethyl cellulose (HEC) and glycerin were added to water and mixed on a mechanical stirrer (ChemLand, Stargard, Poland) for 5 min at stirring speed of 250 rpm. Then, the polymer solution was heated to 60 °C and then cooled to room temperature while constantly stirring. The emulsion (W/O) was prepared according to a modified method of Suñer-Carbó et al. [28]. The emulsion was prepared by a slow addition of the oil phase to the aqueous phase at a temperature of 80 °C under continuous stirring at 300 rpm. The resulting mixture was stirred until a homogeneous emulsion was completely formed. FA and its derivatives dissolved in 96% ethanol were added to the formed and cooled hydrogel or emulsion vehicles. Three hydrogels containing individual ferulic acid derivatives, namely H-GPr[FA], H-LPr[FA], H-PPr[FA], and control containing pure ferulic acid H-[FA] as well as four similar emulsions, namely E-GPr[FA], E-LPr[FA], E-PPr[FA], and E-[FA]. For comparison, the vehicles without addition of active substances were also evaluated (Supplementary Materials S6.). The compositions of the hydrogel and emulsion preparations are specified in Table 1. In addition, the solutions of ferulic acid and its individual derivatives in 70% (*v*/*v*) ethanol were also prepared at the same concentration of 1 g FA per 100 cm^3^.For the ethanol solution, the following abbreviations were used: EtOH-[FA], EtOH-GPr[FA], EtOH-LPr[FA], and EtOH-PPr[FA].

#### Stability of Hydrogels and Emulsion

The stability of all hydrogels and emulsions was tested as in the previous studies [29,30]. Briefly, the stability of all preparations was evaluated by centrifuge test. The vehicle samples (3 g) were centrifuged (MPW-223e, Mechanika Precyzyjna, Warsaw, Poland) at 2320× *g* at 25 °C for 10 min. Moreover, the stability of all preparations was also evaluated using the heating/cooling test: incubation at 45 °C (Drying Oven, DHG-9075A) for 48 h, followed by incubation at 4 °C (in the refrigerator) for 48 h. The test was performed for six cycles. Stability of all preparations kept at heating/cooling condition were confirmed by visual appearance (Supplementary Materials S6).

### 2.5. Antioxidant Activity and Total Polyphenols Content

#### 2.5.1. DPPH Radical Scavenging Assay

To determine the antioxidant capacity of ferulic acid and its derivatives the DPPH method was used [31,32]. The analyses were performed using the Hitachi UV-Vis Spectrophotometer U-5100 at the wavelength λ = 517 nm. Trolox (6-hydroxy-2,5,7,8-tetramethylchroman-2-carboxylic acid) was used as a reference substance. The antioxidant activity was expressed as %RSA and mmol Trolox dm^−3^. First of all, 1% (*m/v*) ethanolic solutions of ferulic acid and its derivatives—GPr[FA], LPr[FA], PPr[FA]—were prepared. The antioxidant activity of the prepared solutions was measured as follows: 2.85 mL 0.3 mmol dm^−3^ ethanolic solution of the DPPH radical of absorbance about 1.000 ± 0.020 at λ = 517 nm was placed in the tube, and an appropriate amount of the ethanolic solution of ferulic acid or its derivative was added to obtain the molar ratio of the compound to DPPH 0.1, 1.0 and 2.0. Blank samples without antioxidant were prepared in the same way. The tubes were wrapped in aluminum foil and were sealed with a stopper and then incubated for 10 min at room temperature. After this time, spectrophotometric measurements were carried out in triplicate. For determination of antioxidant activity of the acceptor fluid after 24 h of permeation and solutions obtained by extraction of the skin obtained after penetration study, 0.15 mL samples of each solutions were separately added to 2.85 mL of DPPH solution and spectrophotometric measurements were performed. The antioxidant activity was expressed as mmol Trolox·dm^−3^.

#### 2.5.2. Total Polyphenols Content Assay

Total polyphenols content in acceptor fluid taken after 24 h of permeation and in solutions after skin extraction was determined by the Folin–Ciocalteu method, as described previously [33]. The antioxidant activity was measured as follows: to 0.15 mL of the sample (acceptor fluid or solution obtained after skin extraction), 0.15 mL of tenfold-diluted Folin–Ciocalteu reagent, 1.35 mL of 0.01 M aqueous sodium carbonate solution, and 1.35 mL of water were added, mixed thoroughly, and incubated for 15 min at room temperature. After this time, the spectrophotometric measurements were carried out at 765 nm. Gallic acid was applied as a standard, and results were expressed as gallic acid equivalents (GA) in mmol GA·dm^−3^.

### 2.6. Skin Permeation Studies

#### 2.6.1. Skin Preparation and Characteristics before Permeation Studies

In the experiment, abdominal porcine skin coming from the local slaughterhouse was used. Pig skin was chosen because of its similar permeability to human skin [34,35]. The fresh abdominal porcine skin was washed several times in PBS buffer pH 7.4. The skin of 0.5 mm in thickness was dermatomed and divided into 2 cm × 2 cm pieces. The skin samples were wrapped in aluminum foil and stored at −20 °C until use, not longer than 3 months. According to Badran et al., this storage time at this temperature is safe to keep skin barrier properties [36]. Immediately before the experiment, the skin samples were slowly thawed at room temperature for 30 min and then were hydrated by PBS pH 7.4 [37,38,39,40]. Such prepared skin was mounted on donor chamber. Only the undamaged skin pieces with an even thickness were chosen for experiments. The integrity of the skin was checked by impedance measurement using an LCRmeter4080 (Conrad Electronic, Hirschau, Germany), operated in parallel mode at an alternating frequency of 120 Hz (error at kΩ values < 0.5%). The tips of measuring probes were immersed in the donor and acceptor chamber containing PBS (pH 7.4), as described previously [41,42]. Only skin samples of impedance > 3 kΩ were applied as such impedance is similar to the electrical resistance of human skin [42].

#### 2.6.2. High-Performance Liquid Chromatography (HPLC)

The concentration of FA and its derivatives in the acceptor fluid and in the fluid obtained by skin extraction after permeation experiment was assessed using liquid chromatography system (Knauer, Berlin, Germany) consisting of a model 2600 UV detector, Smartline model 1050 pump and Smartline model 3950 autosampler. HPLC was controlled by ClarityChrom 2009 software. The detector was operated at 280 nm. The separations were performed on 125 mm × 4 mm ID chromatographic column filled with Hyperisil ODS (C18), particle size 5 µm, in isocratic mode. The mobile phase consisted of acetonitrile, 1% acetic acid, and MeOH (47/47/6 *v*/*v*/*v*) and was pumped at flow rate of 1 mL min^−1^. The column temperature was set at 25 °C, and the injection volume was 20 µL.

#### 2.6.3. Skin Permeation Experiments

The permeation experiments were performed using Franz diffusion cells (Phoenix DB-6, ABL&E-JASCO, Wien, Austria). The diffusion was 1 cm^2^. The volume of the acceptor chamber was 8 mL. This chamber was filled with PBS solution (pH 7.4). During experiment, a constant temperature of 37.0 ± 0.5 °C was maintained in each diffusion unit [43]. The acceptor chamber content was stirred with a magnetic stirring bar at the same speed for all cells. The prepared pig skin was mounted on each donor chamber. As previously mentioned, only undamaged skin pieces with an even thickness were chosen for experiments. Thereafter, the hydrogel or emulsion vehicle/preparation in 1 g portion was put on the skin in donor cells. For comparison, 1 mL ethanolic solution containing either FA or its derivatives was applied to another donor chamber. The amount of the FA and its derivatives applied on the skin expressed as the mass of ferulic acid was 0.01 g and was the same for each carrier. The penetration experiments were carried out for 24 h. The samples were collected after 1 h, 2 h, 3 h, 4 h, 5 h, 6 h, 8 h, and 24 h. Aliquots of the acceptor fluid (0.5 mL) were withdrawn and to each acceptor chamber the same volume of fresh PBS solution was added. The FA and its derivatives’ concentrations in the acceptor fluid were measured by HPLC method (Section 2.6.2). The cumulative mass (µg·cm^−2^) and penetration rate (µg·cm^−2^·h^−1^) were calculated. The acceptor fluid collected after 24 h of permeation was also measured for antioxidant activity and total polyphenol content (as described in Section 2.5.1 and Section 2.5.2).

#### 2.6.4. Accumulation in the Skin

The accumulation of the FA and its derivatives in the skin after 24 h permeation process through the skin were determined using a modification of the methods described by Janus et al. and Taokaew et al. [6,44]. After 24 h of permeation experiment, the skin samples were removed from the Franz diffusion cell, washed three times with 0.5% sodium lauryl sulfate solution and dried at room temperature. Next, after cutting out the diffusion area of each skin sample, the samples were immersed in 2 mL of absolute ethanol and continuously stirred at 300 rpm at room temperature for 24 h. The samples were then centrifuged, and clear solutions were collected and analyzed by HPLC method for the determination of FA and its derivatives. The accumulation of the active substance in the skin was calculated by dividing the amount of the drug remaining in the skin by a mass of skin sample and was expressed in mass active substance per mass of the skin (µg g^−1^). For solution obtained after skin extraction, the antioxidant activity and total polyphenol content were also determined (as described in Section 2.5.1 and Section 2.5.2).

#### 2.6.5. Fluorescent Microscopy 

The skin samples removed from the Franz diffusion cell were fixed in 4% buffered paraformaldehyde for 24 h. Further skin samples were dehydrated in alcohols from 50% to 99.9% and xylene and then were embedded in paraffin blocks. Paraffin blocks were cut on a rotary microtome to 5 μm thick sections and placed on histological slides. For ferulic acid and its derivatives visualization in histological sections, slides were rehydrated starting with xylene and alcohols (99.9–70%) and finished with a deionized water wash. After deparaffinization, samples were mounted with a fluorescent mounting medium. The Neu reagent (2-aminoethyl diphenylborinate 1% in methanol) was used to identify the FA and its derivatives. Sections were scanned with a confocal microscope (FV-1000 Olympus, Hamburg, Germany) with 405 nm diode laser, Olympus IX81 inverted microscope, and FV10-ASW 4.2, Viewer software, Ver.4.2b, Olypmus).

### 2.7. Cytotoxicity of FA and Its Derivatives by Cell Culture Study

Murine fibroblasts (L929) were cultured in a humidified incubator (5% CO_2_, 37 °C) in DMEM low-glucose medium (Sigma-Aldrich Merck Group, St. Louis, MO, USA), supplemented with heat-inactivated fetal bovine serum (FBS, EURx, Gdansk, Poland), L-glutamine (2 mM, Sigma-Aldrich Merck Group, St. Louis, MO, USA) and penicillin/streptomycin (Sigma-Aldrich Merck Group, St. Louis, MO, USA). For cell culture study stock solution of FA and its derivatives were prepared in 70% *v*/*v* ethanol at concentration of 200 mM ([FA, LPr[FA] and PPr[FA]) and 100 mM of GPr[FA] (because of limited solubility). The cell biocompatibility was evaluated using a PrestoBlue HS Cell Viability Reagent (Thermo Fisher Scientific, MA USA) based on the reduction of the resazurin to highly fluorescent resorufin, of which the amount is directly correlated to the number of living cells. The L929 cells were seeded in 96-well black microplates (Greiner, Austria) at a density of 5 × 10^3^ cells/well and allowed to adhere for 24 h. Afterwards, cell culture medium was removed and replaced with 90 µL of the fresh medium containing 0.03125, 0.0625, 0.125, 0.25, 0.5, 1.0, and 2.0 mM (except GPr[FA], where the highest tested concentration in medium was equal 1.0 mM). The final concentrations of ethanol did not exceed 1.0% and its effect on cell viability was also evaluated. The cells without tested compounds were used as control. The interaction of tested compounds without cells and Prestoblue Reagent (blank wells) was excluded. After 24 h of treatment, optical microscopy imaging of L929 cells was performed using Smart Fluorescent Cell Analyzer Microscope JuLi (Seoul, Korea). Next, the PrestoBlue reagent (10 μL) was added to each well and incubated for 30 min. The fluorescence was measured using a spectrophotometric microplate reader (Infinite 200 Pro, Tecan, Männedorf, Switzerland) at ex/em: 560/594 nm. The readings were acquired from at least three independent experiments (each conducted in triplicate) and normalized to the control (100% viability).

### 2.8. Statistical Analysis

Results are presented as the mean ± standard deviation (SD). A one-way analysis of variance (ANOVA) was used. In the case of the cumulative mass after 24 h permeation and the cumulative mass in the skin the significance of differences between individual groups was evaluated with Tukey test (α < 0.05). A cluster analysis was carried out to determine similarities between all compounds tested and all vehicles considering all time points. In the case of the viability of cells, the significance of differences between treated and control cells was evaluated with Student’s *t*-test (*p* < 0.05). Statistical calculations were performed using Statistica 13 PL software (StatSoft, Kraków, Poland).

## 3. Results

### 3.1. Synthesis and Characteristics of Ferulic Acid Derivatives 

The derivatives of ferulic acid and amino acid propyl esters were prepared by a three-step method (Scheme 1). Three amino acids—glycine (G), L-leucine (L) and L-proline (P)—were chosen due to their important role in maintaining the proper skin structure. It is worth noting that the appropriate complex of these amino acids activates the synthesis of collagen types I and III and restores the balance between the loss and the production of this protein. It is known that L-Leu has been used for the attenuation of skin wrinkles in conjunction with Gly and L-Pro [45,46]. Additionally, glycine prevents muscle degeneration because it provides creatinine and participates in the synthesis of erythrocytes, in the supply of amino acids, and in the biosynthesis of glucose and keratin, which are responsible for energy production. The basic function of L-leucine in the body is to build muscle mass, without which we would not only be unable to move but also breathe or pump blood. On the other hand, L-proline is the main component of tissues—it constitutes over 10% of the proteins in our body. It creates 30% of the pool of amino acids that build collagen proteins. Proline is also a component of myofibril contractile proteins [47].

In the first synthesis step (Scheme 1), the hydrochloride of the amino acid propyl ester was prepared by esterification of the proper amino acid with propanol as both reactant and solvent and with TMSCl as chlorinating agent. Next, amino acid ester hydrochloride was neutralized with ammonium hydroxide aqueous solution. In the third step, the equimolar amounts of AAPr and ferulic acid were ground in a mortar for the preparation of amino acid propyl ester ferulates as white solids. All compounds were identified by ^1^H and ^13^C NMR and FT-IR.

**Figure 1:**
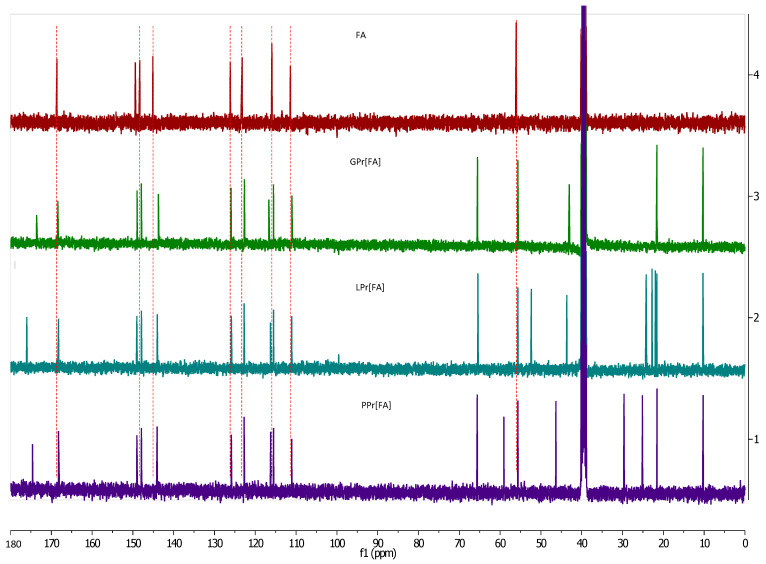
Comparison of ^13^C NMR spectra for ferulic acid and its three derivatives with propyl esters of glycine, L-leucine, and L-proline.

Individual ^1^H NMR, ^13^C NMR, and ^1^H-^13^C HMQC spectra for each compound are included in the Supplementary Materials. The ^13^C NMR spectra of ferulic acid and its derivatives are gathered in Figure 1. In the ^13^C NMR spectra of the derivatives, we can see signals characteristic of the ferulic acid part and amino acid ester moiety. The differences between values of chemical shifts from comparison of ^13^C NMR spectra show that amino acid propyl esters affect the chemical shift of carbon atoms of FA. For almost all carbons, except for the C9 carbon atom, the signals are a little upfield-shifted, which means higher electron density. Only the signal of the C9 carbon, directly bound with a carbonyl carbon, is downfield-shifted and its chemical shift changes from 115.91 ppm for ferulic acid to the range of 116.22–116.66 ppm for FA derivatives. This change indicates a decrease in the shielding of the nucleus of the C9 carbon and a change in the nature of the vicinity carboxyl group to the more ionic. The most distinct changes concern the electronic charge distribution in the region of the double bond between the carbonyl atom and the aromatic ring. The differences between chemical shifts of the C8 and C9 signals are 29.18 ppm for FA and in the range of 27.08–27.84 ppm for FA derivatives. Moreover, in the downfield region in ^13^C NMR spectra of FA derivatives, two carbonyl carbon signals are detected at chemical shifts between 173.61 ppm and 175.98 ppm for the ester moiety of the amino acids and between 168.22 and 168.37 ppm for the carboxyl groups of FA in its derivatives. The latter is a little upfield-shifted compared to this signal for unmodified FA (168.61 ppm).

**Figure 2:**
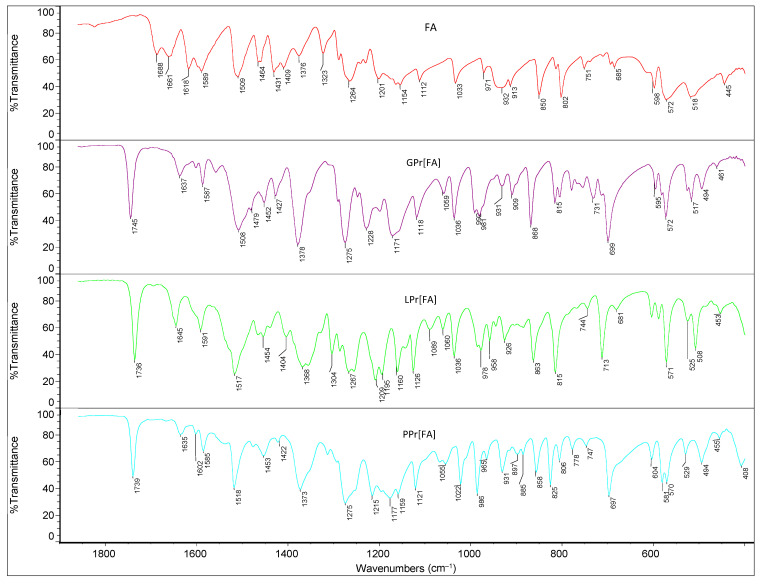
Comparison of FTIR spectra in the wavenumber range 1900–400 cm^−1^ for ferulic acid and its three derivatives with propyl esters of glycine, L-leucine, and L-proline.

The FTIR spectra in the wavenumber range 1900–400 cm^−1^ for the derivatives of ferulic acid and propyl esters of glycine, L-leucine, and L-proline were collated with the spectrum of the parent acid in Figure 2. Copies of FTIR spectra for the individual compounds are available in Supplementary Materials. The characteristic for carboxylic acids, the stretching vibration bands of the carbonyl groups ν(C=O) at 1688 and 1661 cm^−1^ as well as ν(C–O) at 1323 cm^−1^ and ν(O–H)_COOH_ at 3430 cm^−1^, which are strong in the spectrum of FA, disappear in the spectra of FA derivatives. The absence of these bands in the spectra of derivatives proves that new structures were formed. In addition, the formation of carboxylate anions can be evidenced by the appearance of low intensity bands at 1452–1454 cm^−1^ (ν_s_ (COO^−^)) and 1602 cm^−1^ (ν_as_(COO^−^)), characteristic for salts of carboxylic acids. Moreover, olefin C=C stretching vibrations band occurs in the spectrum of FA at 1618 cm^−1^ is shifted toward higher wavenumbers 1635–1645 cm^−1^, in the spectra of FA derivatives. This suggests an increase in electronic charge density around the olefinic double bond for the vicinity of the carboxylate anions. Such a type of shift was also found in the interaction of FA with metals [48]. In addition, the band at 1736–1745 cm^−1^ (ν(C=O)) is characteristic of amino acid alkyl esters groups and is detected in the spectra of FA derivatives but is not in the ferulic acid spectrum. Thus, the characteristic changes in the FTIR spectra of the FA derivatives in comparison with the spectrum of FA indicate the formation of ionic pairs between the ferulate anion and amino acid alkyl ester cation.

The thermal properties for ferulic acid and its derivatives are presented in Table 2. The curves from DSC and thermogravimetric analysis (TG, DTG, c-DTA) are collected in the Supplementary Materials. All obtained derivatives of ferulic acid are solids with melting points lower than for the parent acid (178.15 °C). The lowest melting point (109.76 °C) was determined for combination of FA with L-proline propyl ester, while the highest (152.91 °C) was for glycine propyl ester. From the DSC analysis, it was detected that ferulic acid crystallizes with an exothermic peak at 125.22 °C. However, for FA derivatives, the exothermic crystallization peaks were not detected, and no melting peaks occur on the second heating curve. It can indicate the solidification of the compounds to the amorphous glass.

As is shown in Table 2, all FA derivatives exhibit similar thermal stability, which is lower than for the parent acid. The decomposition of ferulic acid, according to the DTG curve, proceeds in the temperature range of 175–300 °C with the maximum rate of mass loss at 251.2 °C. whereas the main weight loss of the amino acid propyl ester ferulates is recorded in the temperature range of 120–200 °C with the maximum rate of mass loss between 145.3 °C for GPr[FA] and 156.0 °C for LPr[FA]. However, in the range up to 100 °C, which is sufficient for cosmetics formulations, all compounds are thermally stable. 

Wide application of ferulic acid in skin care and cosmetics products is limited by its poor solubility in water and also in oils. In Table 3, the solubility of three modification products of ferulic acid with propyl esters of glycine, L-leucine, and L-proline is demonstrated and compared to the solubility of unmodified FA. All obtained FA derivatives exhibit better solubility in water, expressed in gram of compound or active substance (FA) per liter. The highest solubility is provided by the combination of FA with L-proline propyl ester. When the solubility in water is expressed in g FA dm^−3^, PPr[FA] is 60 times more soluble (42.1828 ± 3.3793) than ferulic acid (0.6729 ± 0.0828). The solubilities of GPr[FA] and LPr[FA] are close to each other and are seven times lower than PPr[FA] but still much better compared to the unmodified acid. The lipid matrix in the outer layer of the skin is crucial for the skin barrier function. However, an effective action of bioactive ingredients involves their dissolution, distribution between vehicle and skin, and diffusion into skin. Therefore, besides solubility in water, the partition coefficient between *n*-octanol and water is the important physical parameter for prediction of transdermal permeation in topical applications. Moreover, both the solubility in water and the partition coefficient can also be crucial for the selection of an appropriate formulation for active ingredients. In Table 3 also shows values of partition coefficients for ferulic acid and three products of its modification. The log P_O/W_ for ferulic acid is 1.643 ± 0.003, which means that almost 98% of ferulic acid is partitioned to the *n*-octanol. The prepared salts of ferulic acid with amino acid propyl esters are characterized by partition coefficient value between 0.447 for PPr[FA] and 0.582 for GPr[FA], which is 3–4 times lower than for unmodified acid. For these values of the partition coefficient, above 70% of the compound is still located in octanol.

### 3.2. Evaluation of Free Radical Scavenging Activity of Ferulic Acid Derivatives

Table 4 presents the antioxidant activity of ferulic acid (FA) and its derivatives GPr[FA], LPr[FA], and PPr[FA] carried out using the DPPH method. Increasing the molar ratio of the respective antioxidant to DPPH from 0.1 to 2.0 increases antioxidant activity from 12.4 ± 0.001 %RSA (0.049 ± 0.001 mmol Trolox dm^−3^) to 86.5 ± 0.001 %RSA (0.763 ± 0.001 mmol Trolox dm^−3^ in the case of FA), from 17.6 ± 0.001 %RSA (0.099 ± 0.001 mmol Trolox dm^−3^) to 86.6 ± 0.001 %RSA (0.764 ± 0.001 mmol Trolox dm^−3^ in the case of GPr[FA]), from 17.6 ± 0.001 %RSA (0.098 ± 0.001 mmol Trolox dm^−3^) to 87.8 ± 0.001 %RSA (0.776 ± 0.001 mmol Trolox dm^−3^ in the case of LPr[FA]), and from 19.3 ± 0.001 %RSA (0.115 ± 0.001 mmol Trolox dm^−3^) to 87.7 ± 0.001 %RSA (0.775 ± 0.001 mmol Trolox dm^−3^ in the case of PPr[FA]); see Table 4. The higher antioxidant activity of FA derivatives compared with unmodified FA is clearly visible in the case of lower concentrations, and the difference disappears for the highest concentration. This proves a more effective free radical scavenging by FA derivatives already at lower concentrations.

### 3.3. Stability of Hydrogels and Emulsion

The penetration tests were carried out using hydrogel and emulsion as vehicles for the FA and its derivatives. After preparing the emulsions and hydrogels with and without active compounds, their stability and separation was tested. Both centrifugation tests and temperature shocking tests demonstrated that control samples and preparations with FA and its derivatives are uniform in color and do not separate (Supplementary Materials S6). 

### 3.4. Skin Penetration Study 

**Figure 3:**
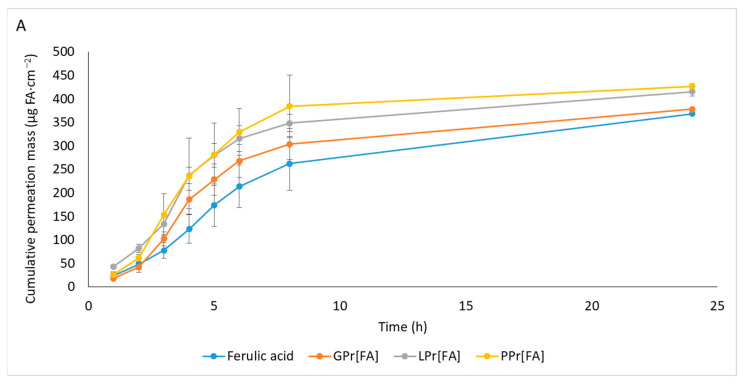

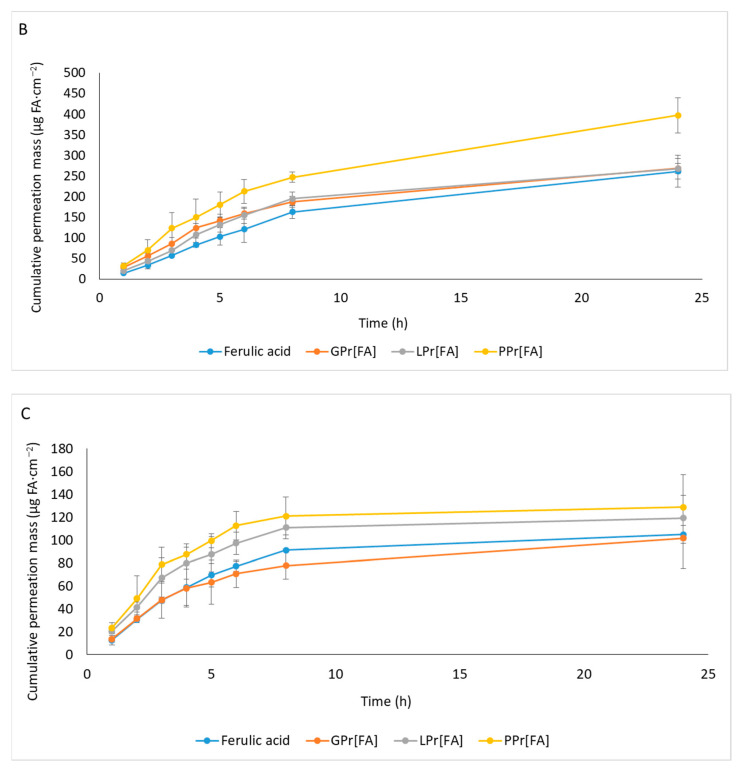
Time course of the cumulative mass of [FA] and its derivatives expressed in µg FA·cm^−2^ during the 24 h permeation from ethanol solution (**A**), hydrogel (**B**), and emulsion (**C**). A constant.

**Figure 4:**
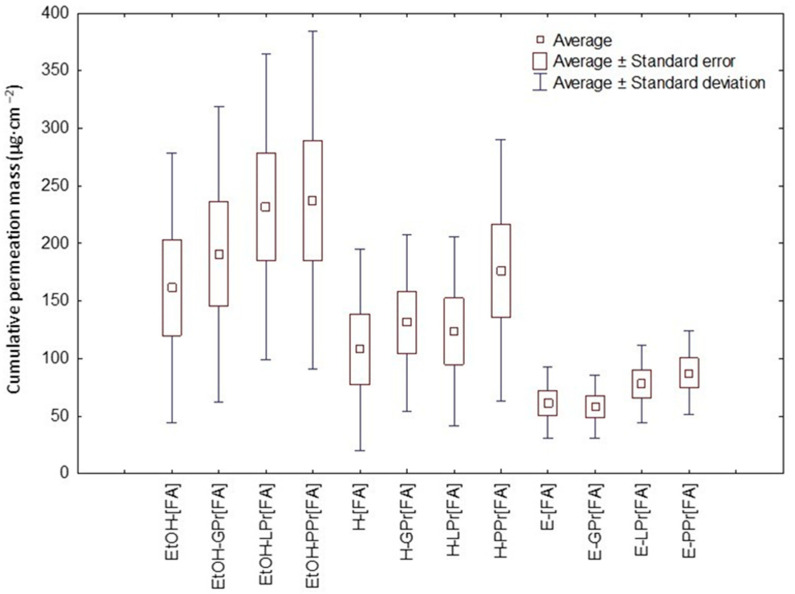
The box—plot of cumulative mass for [FA] and its derivatives after 24 h permeation.

**Figure 5:**
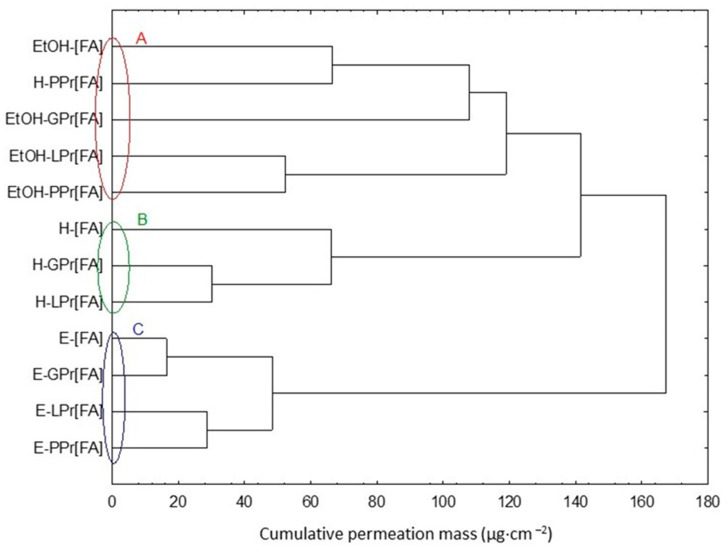
Cluster analysis graph for the mean accumulated mass of [FA] and its derivatives expressed in µg FA·cm^−2^ during the entire 24 h; letters A, B, C mean similar permeation of the analyzed compounds.

In the permeation studies through the skin, the ferulic acid and its derivatives were placed in donor chamber as solution in 70% (*v*/*v*) ethanol and as hydrogel and emulsion preparations in the amount equal to 10 mg expressed as ferulic acid. The cumulative mass of the tested compounds in acceptor fluid, considering all time points, is presented in Figure 3A–C. Moreover, the content of FA and its derivatives in the acceptor fluid collected after 24 h permeation is summarized in Table 5. All analyzed compounds permeated the skin; however, permeation was dependent on the vehicle used and the analyzed compounds. In the case of ethanol solution, the individual compounds permeated in the following order: PPr[FA] > LPr[FA] > GPr[FA] and EtOH-[FA]. In case of this vehicle from among the studied derivatives, PPr[FA] and LPr[FA] permeated to a significantly higher degree than others; the cumulative amounts of substance permeated during the 24 h study were 427.00 ± 4.674 µg FA·cm^−2^ and 415.12 ± 8.71 µg FA·cm^−2^, respectively. A similar result was observed in the case of the hydrogel, where the highest penetration was found for PPr[FA], 396.86 ± 42.13 µg FA·cm^−2^. Other derivatives as well as pure FA penetrated from this vehicle in a significantly smaller amount. Percutaneous permeation of all analyzed compounds from the emulsion was the least effective. The cumulative mass determined in case of this vehicle after 24 h of penetration was also the highest for PPr[FA]—128.80 ± 19.86 µg FA·cm^−2^ (Table 5, Figure 3A–C). In general, the highest permeation from the ethanol solution was observed, then from the hydrogel, and the lowest from the emulsion (Figure 4). This was also confirmed by the cluster analysis graph, where three separate groups with similar permeability can be distinguished (Figure 5).

**Figure 6:**
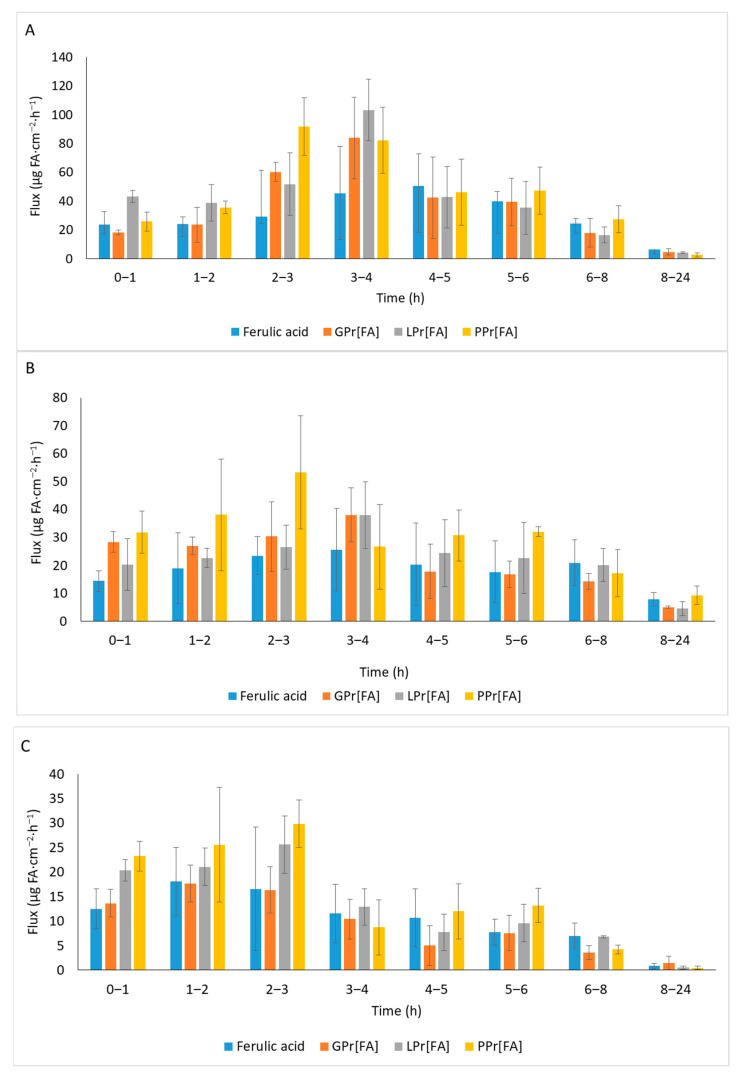
The permeation rate of FA and its derivatives during the 24 h permeation from ethanol solution (**A**), hydrogel (**B**), and emulsion (**C**). α = 0.05 (mean ± SD, n = 3).

The permeation rate determined at each time interval is presented in Figure 6A–C. The highest permeation rate to the acceptor fluid for ethanol solution was observed in samples collected between 2 and 4 h (µg·cm^−2^), while in the case of hydrogels and emulsion this value was between 0 and 3 h of the study.

**Figure 7:**
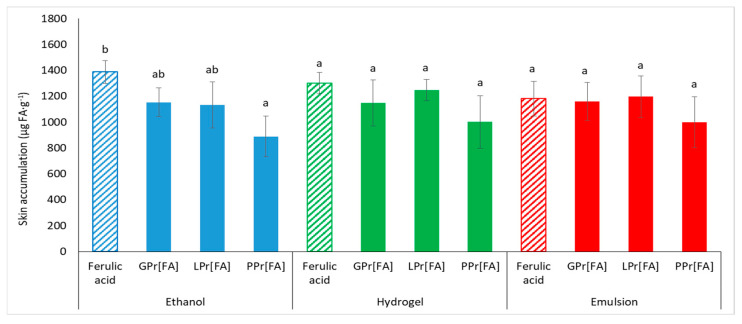
Accumulation in the skin of FA and its derivatives during the 24 h penetration; different letters indicate significant differences between the tested compounds; α = 0.05 (mean ± SD, n = 3). The statistically significant difference was estimated by ANOVA using the Tukey test.

Figure 7 shows the mass of FA and its derivatives accumulated in porcine skin after 24 h of penetration. All the compounds used accumulated in the skin. In general, no significant differences were found in the accumulation of FA and its derivatives in the skin. The exception was PPr[FA], which, after penetration with ethanol solution, accumulated in a significantly smaller amount compared to pure FA. 

**Figure 8:**
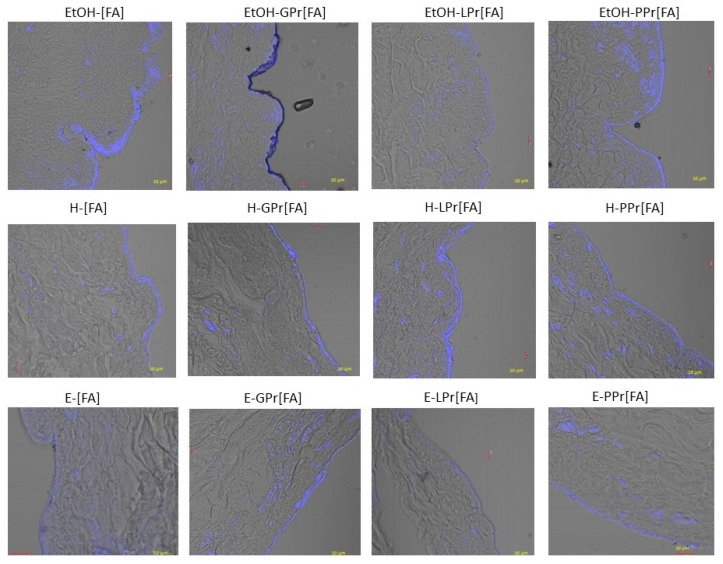
The microscopic images of vertical sections of porcine skin 24 h after applying all the preparations containing FA and its derivatives. The FA and its derivatives are visible mainly in the upper layer of the skin, along the SC (blue color).

Images from transverse sections of pig skin after application of all vehicles are shown in Figure 8. It has been observed that FA and its derivatives strongly accumulate, mainly along the SC of the skin. 

Antioxidant activity and total polyphenol content were evaluated in the samples obtained during the in vitro skin penetration study. The determinations were performed in acceptor fluid collected after 24 h penetration (Table 6) and in the fluid obtained after skin extraction following penetration completion (Table 7). All the tested samples showed antioxidant activity, evaluated by the DPPH method. In the case of permeation experiments of FA and its derivatives from ethanolic solutions as donor, the acceptor fluid collected after the penetration exhibits the antioxidant activity from 0.079 ± 0.010 mmol Trolox dm^−3^ for FA to 0.098 ± 0.011 mmol Trolox dm^−3^ for LPr[FA]. For hydrogels, the values were from 0.063 ± 0.009 mmol Trolox dm^−3^ for LPr[FA] to 0.075 ± 0.012 mmol Trolox dm^−3^ for PPr[FA], whereas in the case of emulsions, the values were from 0.053 ± 0.005 mmol Trolox dm^−3^ for LPr[FA] to 0.061 ± 0.007 mmol trolox dm^−3^ for [PPr[FA]. The content of total polyphenols in the acceptor fluid collected after permeation from the ethanolic solution was the highest for solution of the derivative PPr[FA] and LPr[FA] amounting to 0.122 ± 0.002 and 0.125 ± 0.002 mmol GA dm^−3^, respectively. For the acceptor fluid taken after the hydrogel and emulsion application, the content of total polyphenols was the highest after penetration PPr[FA], i.e., 0.082 ± 0.001 and 0.061 ± 0.002 mmol GA dm^−3^, respectively—Table 6.

The fluid obtained after skin extraction showed high antioxidant activity after each vehicle used. In the case of ethanol solution, the fluid collected after the skin extraction exhibited antioxidant activity from 0.394 ± 0.004 mmol Trolox dm^−3^ for FA to 0.255 ± 0.038 mmol Trolox dm^−3^ for [Leu[FA]. For hydrogels, activities were from 0.381 ± 0.011 mmol Trolox dm^−3^ for FA to 0.298 ± 0.009 mmol Trolox dm^−3^ for LPr[FA], whereas in the case of the emulsions, the values were from 0.300 ± 0.024 mmol Trolox dm^−3^ for [FA] to 0.235 ± 0.037 mmol Trolox dm^−3^ for GPr[FA]. The total content of polyphenols in the fluid skin extraction collected after penetration from the ethanolic solution was significantly the highest for fluids containing FA and derivative GPr[FA], with values of 0.275 ± 0.015 and 0.234 ± 0.004 mmol GA dm^−3^, respectively. A similar tendency was observed when the hydrogel formulations were applied., while in these cases of emulsion, the total content of polyphenols was the highest after penetration of derivative GPr[FA], namely 0.264 ± 0.000 mmol GA dm^−3^; see Table 7.

### 3.5. Cell Culture Study

**Figure 9:**
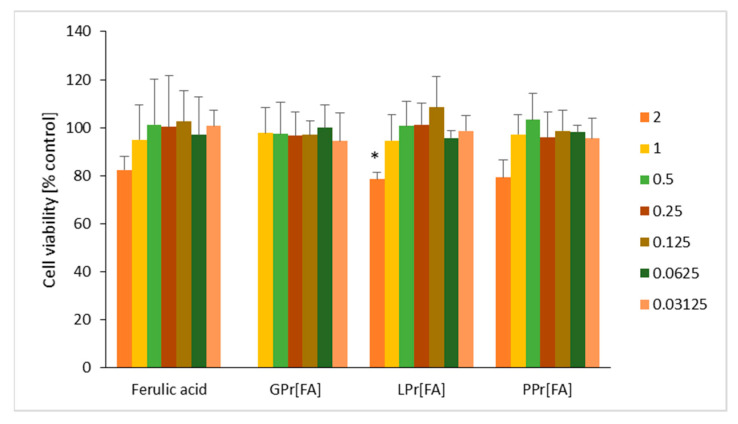
The viability of L929 cells after 24 h exposure to FA and its derivatives, measured by PrestoBlue assay. *—*p* < 0.05 statistical significance between treated and control cells (Student’s *t*-test).

**Figure 10:**
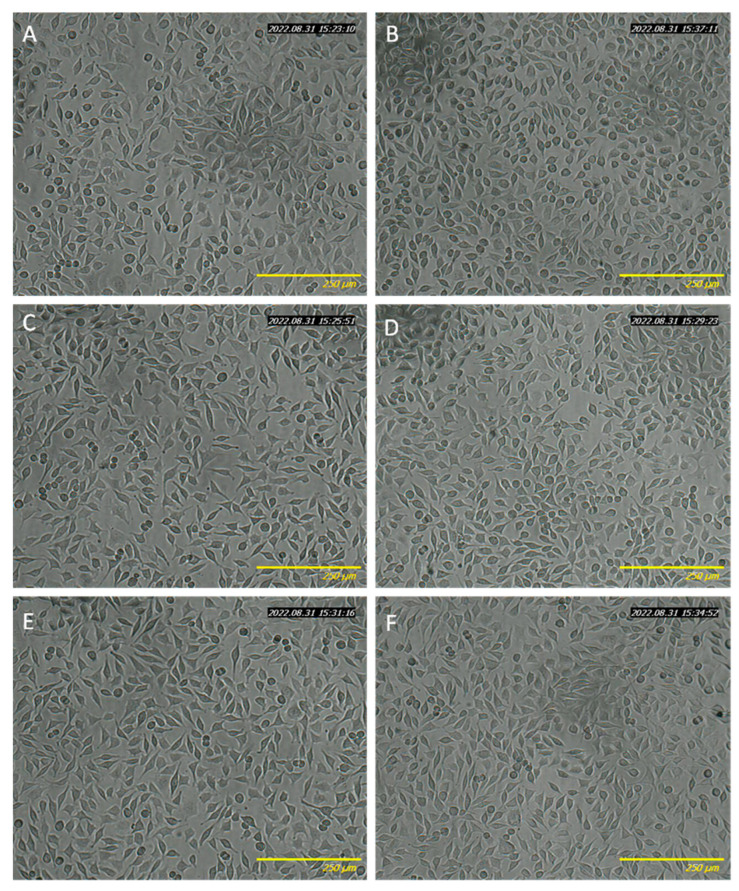
Optical microscopy images of L929 cells after 24 h exposure to: (**A**) control, (**B**) control with 1.0% of ethanol, (**C**) FA (2 mM), (**D**) GPr[FA] (1 mM), (**E**) LPr[FA] (2 mM), (**F**) PPr[FA] (2 mM).

The biocompatibiliy of FA and its derivatives were evaluated using resazurin-based cell viability assays. The L929 cells were treated with increasing doses of FA, GPr[FA], LPr[FA], and PPr[FA] for 24 h. No statistically significant differences in viability between control and FA-treated cells (at any dosage) were observed (Figure 9). Similar activity characterized FA derivatives, except for 2.0 mM of PPr[FA], which decreased L929 viability to 78.56 ± 2.73%. The PrestoBlue results were compared with microscopy imaging, and here no striking differences in cell morphology and density between controls and treated cells were observed (Figure 10).

## 4. Discussion

Ferulic acid is a well-known bioactive substance with multiple functions. It shows its main action as antioxidant and anti-inflammatory factors but also has a protective effect on the kidney as well as the circulatory system [2]. In recent years, there has been a growing interest to apply it as a cosmetics ingredient, which is mainly related to its high antioxidant activity. 

In our study, we attempted the synthesis and evaluation of new ferulic acid derivatives, such as glycine propyl ester ferulate (GPr[FA]), L-leucine propyl ester ferulate (LPr[FA]), and L-proline propyl ester ferulate (PPr[FA]). In the first stage of our study, compounds were identified by NMR, FTIR, and solubility studies. 

The log P_O/W_ for ferulic acid is 1.643 ± 0.0033, which means that almost 98% of ferulic acid is partitioned to *n*-octanol. Similar values of partition coefficient for ferulic acid were determined by other authors, i.e., 1.69 [49] using reversed-phase HPLC analysis and 1.77 [49] and 1.32 ± 0.01 [50] using the shake flask method. The prepared salts of ferulic acid with amino acid propyl esters are distinctly more hydrophilic than the unmodified acid. Although the partition coefficients for the obtained ferulates of amino acid propyl esters are 3–4 times lower than for the unmodified acid, the greater part of these compounds is still partitioned into *n*-octanol, i.e., from 74% for PPr[FA] to 79% for GPr[FA]. Much higher solubility in water than ferulic acid, with a high proportion still partitioning into octanol, may suggest that these compounds easily overcome the lipid organization of the intercellular space of the skin and simultaneously more easily penetrate by intracellular pathway into deeper layers of the skin.

Their antioxidant activity by the DPPH method was also determined. All derivatives were characterized by high antioxidant activity, which was confirmed by other works on other FA derivatives. For example, it was found that sodium ferulate could protect human corneal endothelial cells from the oxidative damage induced by lidocaine, which causes corneal thickening, opacification, and corneal endothelial cell loss [51]. In another study a derivative of FA, hmy-paa (3-(4-hydroxy-3-methoxyphenyl)-N-(1H-pyrazol-3-yl) acrylamide), was demonstrated to selectively inhibit succinate dehydrogenase activity and efficiently abate myocardial cell injury caused by hypoxia [52]. On the other hand, the protective effect of FA derivatives on the skin was presented by Di Domenico et al. In their study, the ferulic acid ethyl ester FAEE protected skin melanocytes from UV-induced oxidative stress and cell damage. Treatment with FAEE reduced the production of ROS in UVB irradiated human melanocytes. FAEE caused induction of heat shock protein 70 (HSP70) and heme oxygenase by a marked suppression of poly (ADP-ribose) polymerase (PARP) activation and a significant suppression of apoptosis. Moreover, FAEE prevented nitric oxide synthase (iNOS) induction, thus suppressing the secondary generation of NO-derived oxidizing agents [20]. 

In our study, we estimated the permeation of pure FA and its derivatives from two popular and frequently used pharmaceutical/cosmetics vehicles and, for comparison, from an ethanol solution. The FA belongs to a large group of polyphenols that are attractive as ingredients in cosmetics formulations as result of their ability to delay the aging process. In addition, it can be used as an antioxidant to prevent damage from ultraviolet (UV) radiation and skin photodamage [17]. However, different factors limit its use, including low solubility and poor skin permeability [53]. Delivery of antioxidants, including FA, via the skin is an attractive alternative to oral dosing, which is associated with many side effects [17]. The permeation of ferulic acid and its derivatives has been investigated earlier by Zhang et al. They analyzed derivatives such as ferulic acid ethyl ether (FAEE), coniferyl aldehyde (CD), coniferyl alcohol (CA), and 3-hydroxy-4-methoxycinnamic acid (HMA) [17]. The penetration of pure ferulic acid, as well as the penetration of this compound from various natural resources, such as propolis, was analyzed more frequently [54,55,56,57,58].

The objective of this study was to examine both transdermal permeation and the local skin concentration of FA and its derivatives. The obtained results showed that derivatives, in particular PPr[FA], exhibited the highest permeation among all compounds. The derivative PPr[FA] permeated more effectively from the ethanol solution and from the hydrogel compared to the unmodified FA in these vehicles. The vehicle used may be of key importance in the penetration of active substances. In the case of topical penetration, it is very important to release the given substances from the preparation form and to reach all layers of the skin as well as the underlying layers [59]. We observed, that skin penetration was dependent on the vehicle used. Generally, the highest penetration of FA and its derivatives was observed from the ethanol solution, the next from the hydrogel, and the lowest from the emulsion. The high permeation from solution in 70% (*v*/*v*) ethanol is evident because such solvent is an optimal vehicle for transepidermal application of the drug [60,61]. Higher ethanol concentration, above 70%, caused dehydration of the skin and decreased the drug penetration [62]. Ethanol is a solvent capable of enhancing drug penetration across the skin. The ethanol “disorders” the intercellular lipid bilayers of the SC [63]. Comparing the cosmetics vehicles used in the presented study, such as hydrogel and emulsion, it was found that all the analyzed compounds penetrated much better from the hydrogel. The hydrogels can be a good vehicle for topically applied drugs [64,65]. Due to their high water content, hydrogels exhibit favorable biocompatibility as well as being easy to apply [29,66,67]. 

The greater permeation of the derivatives was probably also due to their greater solubility. Modification of the substance to increase its solubility is probably one of the reasons to increase its permeation through the skin, which has been shown in previous studies on anti-inflammatory drugs [14,68]. 

Our study also assessed the accumulation of FA and its derivatives in the skin after 24 h penetration. It was observed that all the analyzed compounds accumulate in the skin. No significant differences were found between FA and the derivatives, with the exception of PPr[FA] which, when released from the ethanol solution, accumulated to a lesser extent compared to pure FA. The accumulation of this compound in the upper layers of the skin is desirable due to its UV-protective effect. In the case of a cosmetics preparation, the accumulation of active substances in the skin is beneficial. The accumulated active substances on the skin may have an antioxidant effect for a longer time, showing beneficial anti-aging or anti-inflammatory effects, among others, while in the case of ferulic acid and its derivatives, the photoprotective effect is also important. In our study, the acceptor fluid obtained after 24 h penetration showed antioxidant activity, which could also suggest such action in deeper layers. Photodamage of skin is one of the most frequently occurring dermatological problems. A majority of UVB is absorbed in the epidermis of the skin, but UVA reaches deeper skin layers, such as the dermis, and produces reactive oxygen species (ROS). Excessive amounts of ROS could be the cause of the aging, inflammation, and other severe problems such as melanoma. Application of exogenous antioxidants to the skin can be counteracted [69,70]. The FA is responsible for chelating protonated metal ions, such as Cu(II) or Fe(II). The mechanism of antioxidative activity of FA involves their ability to form stable phenoxyl radicals by the reaction of the radical molecule with the molecule of antioxidant. It makes it difficult to initiate a complex reaction cascade leading to the generation of free radicals. This compound may also act as hydrogen donor, giving atoms directly to the radicals [1].

In our study, the activity of FA amino acids derivatives in the cell culture conditions was also assessed. The biocompatibility of [FA], GPr[FA], LPr[FA], and PPr[FA] were tested in vitro using the L929 murine fibroblasts as model cells in a cytotoxicity study. Our data confirmed the biocompatibility of [FA] and PPr[FA] at a concentration ranging from 0.03125 to 2.0 mM, and for GPr[FA] and LPr[FA], the range was from 0.03125 to 1.0 mM. Similar safety of FA was confirmed by Borges et al., who tested the effect of various isothiocyanates and phenolic compounds on the viability of L929 cells. Namely, after 72 h of treatment, FA, at the concentration of 500 μg mL^−1^ i 1000 μg mL^−1^ (corresponding to 2.57 mM and 5.15 mM), reduced cell viability to 67% and 28%, respectively [71]. In another study performed on L929 cells, [FA] (2.5–40 mg L^−1^, corresponding to 3 µM–206 µM) after 24 h incubation, decreased cell viability to 80% only at the highest tested concentration (according to Trypan blue results) and has no effect on cell viability/proliferation regarding the MTT assay. Surprisingly, both assays revealed a significant effect of FA on intestinal cells (Caco2) at a concentration of 10 and 20 mg L^−1^, but not 40 mg L^−1^ [72]. Therefore, Li et al. [2] suggested that FA has cell-dependent toxicity and should be used with caution [2]. In turn, interesting results where reached by Choi et al. [73], who showed that FA (500 μg mL^−1^) significantly decreased the viability of cancer cells (HepG2) but did not cause any toxicity on normal NIH-3T3 and 3T3-L1 cells [73]. Two independent studies have shown that the antitumor properties of FA against osteosarcoma cells [74] and cervical carcinoma cells [75] were associated with G0/G1 cell-cycle arrest and induction of apoptosis. Furthermore, FA not only decreased viability and increased apoptosis of a breast cancer cell line (MDA-MB-231) but also suppressed breast cancer migration and metastasis in vivo [76].

## 5. Conclusions

The study presents the synthesis, identification, and characterization of three new FA amino acid derivatives, namely glycine propyl ester ferulate (GPr[FA]), L-leucine propyl ester ferulate (LPr[FA]) and L-proline propyl ester ferulate (PPr[FA]). The obtained compounds showed significantly improved solubility in water compared to unmodified ferulic acid. Additionally, the FA derivatives usually provided increased permeability of the active substance through porcine skin. The highest permeability was observed for PPr[FA] from ethanolic solution and hydrogel formulation. Furthermore, the high antioxidant activity of both the acceptor fluid collected after 24 h of permeation and the fluid obtained after skin extraction were demonstrated for all compounds. The total polyphenol content was usually higher in the acceptor fluid for the best permeating PPr[FA], while in the skin extracts it was least permeating for unmodified FA and GPr[FA]. The more effective percutaneous permeation of PPr[FA] may increase the probability of broader antioxidant action in deeper tissues. Interestingly, all the analyzed compounds accumulated in the skin, usually in its upper layer or just below it. This is also very beneficial because the accumulated active substances in the skin may have an antioxidant effect for a longer time, showing beneficial effects such as antiaging, anti-inflammatory, or photoprotective effects, among others. Therefore, the derivatives of FA and amino acid propyl esters after topical application can be an interesting alternative in skin protection and also systemic action. In addition to providing the active substance of ferulic acid, the presented derivatives are also a source of amino acids (L-Pro, L-Leu, Gly), which are important for the structure of the skin, for both the construction and strengthening of proteins and myofibril contractile proteins. Thus, they can enhance antiaging effects and attenuate skin wrinkles. It is worth emphasizing that ferulic acid and its amino acid derivatives have a little effect on the viability of L929 murine fibroblasts, which decreased to 80% for all compounds only at the highest concentration.

## Figures and Tables

**Table 1:** Composition of the prepared hydrogels and emulsions used in the penetration study.

Ingredient	Emulsion	Hydrogel
FA or its derivatives *	1 *	1 *
HEC	-	3
Glycerin	10	10
Biobase	6	-
Grape seed oil	20	-
Beeswax	7	-
Ethanol 96%	1	1
Water up to	100	100

Amount of the components is expressed in g; HEC—hydroxyethylcellulose; * the amount of added FA derivatives was converted into FA so that the concentration of the active substance (FA) in all final preparations was 1 g FA per 100 g.

**Table 2:** The properties of ferulic acid and amino acid propyl ester ferulates.

Compound	Molar Mass (g mol^−1^)	T_m_ (°C)	T_c_ (°C)	T_d onset_ (°C)	T_d max_ (°C)	${\left[\alpha \right]}_{D}^{20}$
Ferulic acid	194.18	178.15	125.22	217.0	251.2	na
GPr[FA]	311.33	152.91	nd	129.6	145.3	na
LPr[FA]	367.44	115.27	nd	126.0	156.0	+9.6
PPr[FA]	351.39	109.76	nd	122.8	148.7	−29.8

T_m_—melting point peak based on DSC measurement; T_c_—crystallization peak temperature; T_d _onset__—the onset of decomposition; T_d max_—temperature of the fastest mass loss of the sample; [α]_λ_^T^ specific rotation measured for 1% (*m*/*v*) solution of compound in 70% ethanol; nd—not defined; na—not applicable.

**Table 3:** The solubility in water at the temperature of 25 °C and *n*-octanol/water partition coefficient and log P_O/W_ of ferulic acid and its derivatives with amino acid propyl esters.

Compound	Solubility g dm^−3^	Solubility g FA dm^−3^	log P_O/W_
Ferulic acid	0.6729 ± 0.0828	0.6729 ± 0.0828	1.643 ± 0.003
GPr[FA]	9.5816 ± 1.5818	5.9760 ± 0.9866	0.582 ± 0.019
LPr[FA]	11.3416 ± 1.4321	5.9937 ± 0.7568	0.454 ± 0.009
PPr[FA]	76.3345 ± 6.1153	42.1828 ± 3.3793	0.447 ± 0.022

**Table 4:** The antioxidant activity of the FA and its derivatives; different letters indicate significant differences between the tested compounds. α = 0.05. mean ± SD. n = 3. The statistically significant difference was estimated by ANOVA using the Tukey test.

	Antioxidant Activity:							
The Molar Ratio Antioxidant:DPPH	FA		GPr[FA]		LPr[FA]		PPr[FA]	
	%RSA	mmol Trolox dm^−3^	%RSA	mmol Trolox dm^−3^	%RSA	mmol Trolox dm^−3^	%RSA	mmol Trolox dm^−3^
0.1	12.4 ± 0.001 a	0.049 ± 0.001 a	17.6 ± 0.001 a	0.099 ± 0.001 a	17.6 ± 0.001 a	0.098 ± 0.001 a	19.3 ± 0.001 a	0.115 ± 0.001 a
1.0	73.2 ± 0.001 b	0.635 ± 0.001 b	78.0 ± 0.001 b	0.681 ± 0.001 b	79.5 ± 0.001 b	0.696 ± 0.001 b	79.6 ± 0.001 b	0.697 ± 0.001 b
2.0	86.5 ± 0.001 b	0.763 ± 0.001 b	86.6 ± 0.001 b	0.764 ± 0.001 b	87.8 ± 0.001 b	0.776 ± 0.001 b	87.7 ± 0.001 b	0.775 ± 0.001 b

**Table 5:** Cumulated mass for FA and its derivatives; different letters indicate significant differences between the tested compounds, α = 0.05, mean ± SD, n = 3. The statistically significant difference was estimated by ANOVA using the Tukey test.

Cumulative Permeation Mass (µg FA·cm^−2^)	
Ethanol	
FA	368.57 ± 2.94 a
GPr[FA]	378.26 ± 2.29 a
LPr[FA]	415.12 ± 8.71 b
PPr[FA]	427.00 ± 4.67 b
Hydrogel	
FA	289.36 ± 38.50 a
GPr[FA]	268.16 ± 12.71 a
LPr[FA]	267.48 ± 24.57 a
PPr[FA]	396.86 ± 42.13 b
Emulsion	
FA	104.93 ± 7.63 a
GPr[FA]	101.70 ± 26.47 a
LPr[FA]	119.29 ± 19.72 a
PPr[FA]	128.80 ± 19.86 a

**Table 6:** The antioxidant activity of the acceptor fluid collected after 24 h penetration; different letters indicate significant differences between the tested compounds, α = 0.05, mean ± SD, n = 3. The statistically significant difference was estimated by ANOVA using the Tukey test.

Compounds	DPPH mmol Trolox dm^−3^	Total Polyphenol mmol GA dm^−3^
Ethanol		
FA	0.079 ± 0.010 a	0.097 ± 0.002 a
GPr[FA]	0.097 ± 0.021 b	0.115 ± 0.001 b
LPr[FA]	0.098 ± 0.011 a	0.122 ± 0.002 c
PPr[FA]	0.094 ± 0.002 a	0.125 ± 0.001 c
Hydrogel		
FA	0.067 ± 0.004 a	0.059 ± 0.003 a
GPr[FA]	0.069 ± 0.002 a	0.064 ± 0.001 b
LPr[FA]	0.063 ± 0.009 a	0.057 ± 0.002 a
PPr[FA]	0.075 ± 0.012 a	0.082 ± 0.001 c
Emulsion		
FA	0.057 ± 0.004 a	0.047 ± 0.006 a
GPr[FA]	0.059 ± 0.010 a	0.051 ± 0.001 ab
LPr[FA]	0.053 ± 0.005 a	0.045 ± 0.004 a
PPr[FA]	0.061 ± 0.007 a	0.061 ± 0.002 b

**Table 7:** The antioxidant activity of the solution obtained after skin extraction; different letters indicate significant differences between the tested compounds, α = 0.05, mean ± SD, n = 3. The statistically significant difference was estimated by ANOVA using the Tukey test.

Compounds	DPPH mmol Trolox dm^−3^	Total Polyphenol mmol GA dm^−3^
Ethanol		
FA	0.394 ± 0.004 b	0.275 ± 0.015 c
GPr[FA]	0.257 ± 0.026 a	0.234 ± 0.002 b
LPr[FA]	0.255 ± 0.038 a	0.199 ± 0.004 a
PPr[FA]	0.259 ± 0.007 a	0.193 ± 0.005 a
Hydrogel		
FA	0.381 ± 0.011 b	0.289 ± 0.006 c
GPr[FA]	0.298 ± 0.009 a	0.248 ± 0.010 b
LPr[FA]	0.320 ± 0.030 ab	0.233 ± 0.008 b
PPr[FA]	0.351 ± 0.036 ab	0.201 ± 0.007 a
Emulsion		
FA	0.300 ± 0.024 b	0.202 ± 0.012 a
GPr[FA]	0.235 ± 0.037 a	0.264 ± 0.000 b
LPr[FA]	0.257 ± 0.014 ab	0.219 ± 0.003 a
PPr[FA]	0.198 ± 0.006 a	0.208 ± 0.021 a

## Data Availability

Not applicable.

## Supplementary Materials

The following supporting information can be downloaded at: https://www.mdpi.com/article/10.3390/pharmaceutics15010117/s1, S1: Copies of 1H and 13C NMR of ferulic acid; S2: Copies of 1H NMR, 13C NMR and 1H-13C HMQC spectra of amino acid propyl ester ferulates; S3: FTIR spectra of ferulic acid and amino acid propyl ester ferulates; S4: DSC curves for ferulic acid and amino acid propyl ester ferulates; S5: Curves from thermogravimetric analysis of ferulic acid and amino acid; S6: Photographs of hydrogels and emulsions preparations.

## References

[1] Zduńska K., Dana A., Kolodziejczak A., Rotsztejn H. (2018). Antioxidant Properties of Ferulic Acid and Its Possible Application. Skin Pharmacol. Physiol..

[2] Li D., Rui Y., Guo S., Luan F., Liu R., Zeng N. (2021). Ferulic Acid: A Review of Its Pharmacology, Pharmacokinetics and Derivatives. Life Sci..

[3] Turkez H., Arslan M.E., Barboza J.N., Kahraman C.Y., de Sousa D.P., Mardinoğlu A. (2022). Therapeutic Potential of Ferulic Acid in Alzheimer’s Disease. Curr. Drug Deliv..

[4] Zduńska-Pęciak K., Kołodziejczak A., Rotsztejn H. (2022). Two Superior Antioxidants: Ferulic Acid and Ascorbic Acid in Reducing Signs of Photoaging—A Split-face Comparative Study. Dermatol. Ther..

[5] Maya-Cano D.A., Arango-Varela S., Santa-Gonzalez G.A. (2021). Phenolic Compounds of Blueberries (Vaccinium Spp) as a Protective Strategy against Skin Cell Damage Induced by ROS: A Review of Antioxidant Potential and Antiproliferative Capacity. Heliyon.

[6] Nowak A., Zagórska-Dziok M., Ossowicz-Rupniewska P., Makuch E., Duchnik W., Kucharski Ł., Adamiak-Giera U., Prowans P., Czapla N., Bargiel P. (2021). *Epilobium angustifolium* L. Extracts as Valuable Ingredients in Cosmetic and Dermatological Products. Molecules.

[7] Zagórska-Dziok M., Ziemlewska A., Bujak T., Nizioł-Łukaszewska Z., Hordyjewicz-Baran Z. (2021). Cosmetic and Dermatological Properties of Selected Ayurvedic Plant Extracts. Molecules.

[8] Tsay G.J., Lin S.-Y., Li C.-Y., Mau J.-L., Tsai S.-Y. (2021). Comparison of Single and Combined Use of Ergothioneine, Ferulic Acid, and Glutathione as Antioxidants for the Prevention of Ultraviolet B Radiation-Induced Photoaging Damage in Human Skin Fibroblasts. Processes.

[9] Kaur R., Sood A., Lang D.K., Arora R., Kumar N., Diwan V., Saini B. (2022). Natural Products as Sources of Multitarget Compounds: Advances in the Development of Ferulic Acid as Multitarget Therapeutic. Curr. Top. Med. Chem..

[10] El-Gogary R.I., Nasr M., Rahsed L.A., Hamzawy M.A. (2022). Ferulic Acid Nanocapsules as a Promising Treatment Modality for Colorectal Cancer: Preparation and in Vitro/in Vivo Appraisal. Life Sci..

[11] Nowak A., Cybulska K., Makuch E., Kucharski Ł., Różewicka-Czabańska M., Prowans P., Czapla N., Bargiel P., Petriczko J., Klimowicz A. (2021). In Vitro Human Skin Penetration, Antioxidant and Antimicrobial Activity of Ethanol-Water Extract of Fireweed (*Epilobium angustifolium* L.). Molecules.

[12] Ossowicz-Rupniewska P., Rakoczy R., Nowak A., Konopacki M., Klebeko J., Świątek E., Janus E., Duchnik W., Wenelska K., Kucharski Ł. (2021). Transdermal Delivery Systems for Ibuprofen and Ibuprofen Modified with Amino Acids Alkyl Esters Based on Bacterial Cellulose. IJMS.

[13] Ossowicz-Rupniewska P., Nowak A., Klebeko J., Janus E., Duchnik W., Adamiak-Giera U., Kucharski Ł., Prowans P., Petriczko J., Czapla N. (2021). Assessment of the Effect of Structural Modification of Ibuprofen on the Penetration of Ibuprofen from Pentravan^®^ (Semisolid) Formulation Using Human Skin and a Transdermal Diffusion Test Model. Materials.

[14] Klebeko J., Ossowicz-Rupniewska P., Nowak A., Janus E., Duchnik W., Adamiak-Giera U., Kucharski Ł., Prowans P., Petriczko J., Czapla N. (2021). Permeability of Ibuprofen in the Form of Free Acid and Salts of L-Valine Alkyl Esters from a Hydrogel Formulation through Strat-M^TM^ Membrane and Human Skin. Materials.

[15] Murphy B., Grimshaw S., Hoptroff M., Paterson S., Arnold D., Cawley A., Adams S.E., Falciani F., Dadd T., Eccles R. (2022). Alteration of Barrier Properties, Stratum Corneum Ceramides and Microbiome Composition in Response to Lotion Application on Cosmetic Dry Skin. Sci. Rep..

[16] Janus E., Ossowicz P., Klebeko J., Nowak A., Duchnik W., Kucharski Ł., Klimowicz A. (2020). Enhancement of Ibuprofen Solubility and Skin Permeation by Conjugation with L-Valine Alkyl Esters. RSC Adv..

[17] Zhang L.-W., Al-Suwayeh S.A., Hsieh P.-W., Fang J.-Y. (2010). A Comparison of Skin Delivery of Ferulic Acid and Its Derivatives: Evaluation of Their Efficacy and Safety. Int. J. Pharm..

[18] Li J., Sha Y. (2008). A Convenient Synthesis of Amino Acid Methyl Esters. Molecules.

[19] Pei K., Ou J., Huang C., Ou S. (2015). Derivatives of Ferulic Acid: Structure, Preparation and Biological Activities. Annu. Res. Rev. Biol..

[20] Di Domenico F., Di Domenico F., Perluigi M., Foppoli C., Blarzino C., Coccia R., De Marco F., Butterfield D.A., Cini C. (2009). Protective Effect of Ferulic Acid Ethyl Ester against Oxidative Stress Mediated by UVB Irradiation in Human Epidermal Melanocytes. Free. Radic. Res..

[21] Shukla D., Nandi N.K., Singh B., Singh A., Kumar B., Narang R.K., Singh C. (2022). Ferulic Acid-Loaded Drug Delivery Systems for Biomedical Applications. J. Drug Deliv. Sci. Technol..

[22] Sintra T.E., Luís A., Rocha S.N., Lobo Ferreira A.I.M.C., Gonçalves F., Santos L.M.N.B.F., Neves B.M., Freire M.G., Ventura S.P.M., Coutinho J.A.P. (2015). Enhancing the Antioxidant Characteristics of Phenolic Acids by Their Conversion into Cholinium Salts. ACS Sustain. Chem. Eng..

[23] Demurtas M., Onnis V., Zucca P., Rescigno A., Lachowicz J.I., De Villiers Engelbrecht L., Nieddu M., Ennas G., Scano A., Mocci F. (2021). Cholinium-Based Ionic Liquids from Hydroxycinnamic Acids as New Promising Bioactive Agents: A Combined Experimental and Theoretical Investigation. ACS Sustain. Chem. Eng..

[24] Caparica R., Júlio A., Baby A., Araújo M., Fernandes A., Costa J., Santos de Almeida T. (2018). Choline-Amino Acid Ionic Liquids as Green Functional Excipients to Enhance Drug Solubility. Pharmaceutics.

[25] Świątek E., Ossowicz-Rupniewska P., Janus E., Nowak A., Sobolewski P., Duchnik W., Kucharski Ł., Klimowicz A. (2021). Novel Naproxen Salts with Increased Skin Permeability. Pharmaceutics.

[26] Ossowicz-Rupniewska P., Klebeko J., Świątek E., Bilska K., Nowak A., Duchnik W., Kucharski Ł., Struk Ł., Wenelska K., Klimowicz A. (2022). Influence of the Type of Amino Acid on the Permeability and Properties of Ibuprofenates of Isopropyl Amino Acid Esters. IJMS.

[27] Zagórska-Dziok M., Bujak T., Ziemlewska A., Nizioł-Łukaszewska Z. (2021). Positive Effect of *Cannabis sativa* L. Herb Extracts on Skin Cells and Assessment of Cannabinoid-Based Hydrogels Properties. Molecules.

[28] Suñer-Carbó J., Calpena-Campmany A., Halbaut-Bellowa L., Clares-Naveros B., Rodriguez-Lagunas M., Barbolini E., Zamarbide-Losada J., Boix-Montañés A. (2019). Biopharmaceutical Development of a Bifonazole Multiple Emulsion for Enhanced Epidermal Delivery. Pharmaceutics.

[29] Nowak A., Zagórska-Dziok M., Perużyńska M., Cybulska K., Kucharska E., Ossowicz-Rupniewska P., Piotrowska K., Duchnik W., Kucharski Ł., Sulikowski T. (2022). Assessment of the Anti-Inflammatory, Antibacterial and Anti-Aging Properties and Possible Use on the Skin of Hydrogels Containing *Epilobium angustifolium* L. Extracts. Front Pharmacol..

[30] Nowak A., Duchnik W., Makuch E., Kucharski Ł., Ossowicz-Rupniewska P., Cybulska K., Sulikowski T., Moritz M., Klimowicz A. (2021). *Epilobium angustifolium* L. Essential Oil—Biological Activity and Enhancement of the Skin Penetration of Drugs—In Vitro Study. Molecules.

[31] Makuch E., Nowak A., Günther A., Pełech R., Kucharski Ł., Duchnik W., Klimowicz A. (2020). Enhancement of the Antioxidant and Skin Permeation Properties of Eugenol by the Esterification of Eugenol to New Derivatives. AMB Expr..

[32] Brand-Williams W., Cuvelier M.E., Berset C. (1995). Use of a Free Radical Method to Evaluate Antioxidant Activity. LWT—Food Sci. Technol..

[33] Nowak A., Klimowicz A., Duchnik W., Kucharski Ł.L., Florkowska K., Muzykiewicz A., Wira D., Zielonkabrzezicka J., Siedłowska A., Nadarzewska K. (2020). Application of Green-Extraction Technique to Evaluate of Antioxidative Capacity of Wild Population of Fireweed (*Epilobium angustifolium*). Herba Pol..

[34] Khiao In M., Richardson K.C., Loewa A., Hedtrich S., Kaessmeyer S., Plendl J. (2019). Histological and Functional Comparisons of Four Anatomical Regions of Porcine Skin with Human Abdominal Skin. Anat. Histol. Embryol..

[35] Jacobi U., Kaiser M., Toll R., Mangelsdorf S., Audring H., Otberg N., Sterry W., Lademann J. (2007). Porcine Ear Skin: An in Vitro Model for Human Skin. Skin Res. Technol..

[36] Badran M.M., Kuntsche J., Fahr A. (2009). Skin Penetration Enhancement by a Microneedle Device (Dermaroller^®^) in Vitro: Dependency on Needle Size and Applied Formulation. Eur. J. Pharm. Sci..

[37] Haq A., Goodyear B., Ameen D., Joshi V., Michniak-Kohn B. (2018). Strat-M^®^ Synthetic Membrane: Permeability Comparison to Human Cadaver Skin. Int. J. Pharm..

[38] Kuntsche J., Bunjes H., Fahr A., Pappinen S., Rönkkö S., Suhonen M., Urtti A. (2008). Interaction of Lipid Nanoparticles with Human Epidermis and an Organotypic Cell Culture Model. Int. J. Pharm..

[39] Simon A., Amaro M.I., Healy A.M., Cabral L.M., de Sousa V.P. (2016). Comparative Evaluation of Rivastigmine Permeation from a Transdermal System in the Franz Cell Using Synthetic Membranes and Pig Ear Skin with in Vivo-in Vitro Correlation. Int. J. Pharm..

[40] Noreen S., Pervaiz F., Ashames A., Buabeid M., Fahelelbom K., Shoukat H., Maqbool I., Murtaza G. (2021). Optimization of Novel Naproxen-Loaded Chitosan/Carrageenan Nanocarrier-Based Gel for Topical Delivery: Ex Vivo, Histopathological, and In Vivo Evaluation. Pharmaceuticals.

[41] Kopečná M., Macháček M., Nováčková A., Paraskevopoulos G., Roh J., Vávrová K. (2019). Esters of Terpene Alcohols as Highly Potent, Reversible, and Low Toxic Skin Penetration Enhancers. Sci. Rep..

[42] Davies D.J., Ward R.J., Heylings J.R. (2004). Multi-Species Assessment of Electrical Resistance as a Skin Integrity Marker for in Vitro Percutaneous Absorption Studies. Toxicol. Vitr..

[43] Bertges F.S., da Penha Henriques do Amaral M., Rodarte M.P., Vieira Fonseca M.J., Sousa O.V., Pinto Vilela F.M., Alves M.S. (2020). Assessment of Chemical Changes and Skin Penetration of Green Arabica Coffee Beans Biotransformed by Aspergillus Oryzae. Biocatal. Agric. Biotechnol..

[44] Taokaew S., Nunkaew N., Siripong P., Phisalaphong M. (2014). Characteristics and Anticancer Properties of Bacterial Cellulose Films Containing Ethanolic Extract of Mangosteen Peel. J. Biomater. Sci. Polym. Ed..

[45] Albaugh V.L., Mukherjee K., Barbul A. (2017). Proline Precursors and Collagen Synthesis: Biochemical Challenges of Nutrient Supplementation and Wound Healing. J. Nutr..

[46] Yamane T., Morioka Y., Kitaura Y., Iwatsuki K., Shimomura Y., Oishi Y. (2018). Branched-Chain Amino Acids Regulate Type I Tropocollagen and Type III Tropocollagen Syntheses via Modulation of MTOR in the Skin. Biosci. Biotechnol. Biochem..

[47] Wu G., Bazer F.W., Burghardt R.C., Johnson G.A., Kim S.W., Knabe D.A., Li P., Li X., McKnight J.R., Satterfield M.C. (2011). Proline and Hydroxyproline Metabolism: Implications for Animal and Human Nutrition. Amino Acids.

[48] Kalinowska M., Piekut J., Bruss A., Follet C., Sienkiewicz-Gromiuk J., Świsłocka R., Rzączyńska Z., Lewandowski W. (2014). Spectroscopic (FT-IR, FT-Raman, 1H, 13C NMR, UV/VIS), Thermogravimetric and Antimicrobial Studies of Ca(II), Mn(II), Cu(II), Zn(II) and Cd(II) Complexes of Ferulic Acid. Spectrochim. Acta Part A Mol. Biomol. Spectrosc..

[49] Shi Z., He J., Yao T., Chang W. (2004). RP-HPLC Determination of Octanol–Water Partition Coefficients for Bioactive Compounds from Chinese Herbal Medicines. J. Liq. Chromatogr. Relat. Technol..

[50] Vilas-Boas S.M., Cordova I.W., Kurnia K.A., Almeida H.H.S., Gaschi P.S., Coutinho J.A.P., Pinho S.P., Ferreira O. (2022). Comparison of Two Computational Methods for Solvent Screening in Countercurrent and Centrifugal Partition Chromatography. J. Chromatogr. A.

[51] Jiang G., Fan T. (2018). Sodium Ferulate Attenuates Lidocaine-Induced Corneal Endothelial Impairment. Oxidative Med. Cell. Longev..

[52] Wang F., Peng Q., Liu J., Alolga R.N., Zhou W. (2019). A Novel Ferulic Acid Derivative Attenuates Myocardial Cell Hypoxia Reoxygenation Injury through a Succinate Dehydrogenase Dependent Antioxidant Mechanism. Eur. J. Pharmacol..

[53] Oliveira A.L.S., Valente D., Moreira H.R., Pintado M., Costa P. (2022). Effect of Squalane-Based Emulsion on Polyphenols Skin Penetration: Ex Vivo Skin Study. Colloids Surf. B Biointerfaces.

[54] Sauce R., de Oliveira Pinto C.A.S., Velasco M.V.R., Rosado C., Baby A.R. (2021). Ex Vivo Penetration Analysis and Anti-Inflammatory Efficacy of the Association of Ferulic Acid and UV Filters. Eur. J. Pharm. Sci..

[55] Chen M., Liu X., Fahr A. (2010). Skin Delivery of Ferulic Acid from Different Vesicular Systems. J. Biomed. Nanotechnol..

[56] Das S., Wong A.B.H. (2020). Stabilization of Ferulic Acid in Topical Gel Formulation via Nanoencapsulation and PH Optimization. Sci. Rep..

[57] Saija A. (2000). In Vitro and in Vivo Evaluation of Caffeic and Ferulic Acids as Topical Photoprotective Agents. Int. J. Pharm..

[58] Žilius M., Ramanauskienė K., Briedis V. (2013). Release of Propolis Phenolic Acids from Semisolid Formulations and Their Penetration into the Human Skin In Vitro. Evid. -Based Complement. Altern. Med..

[59] Ossowicz-Rupniewska P., Bednarczyk P., Nowak M., Nowak A., Duchnik W., Kucharski Ł., Rokicka J., Klimowicz A., Czech Z. (2021). Sustainable UV-Crosslinkable Acrylic Pressure-Sensitive Adhesives for Medical Application. Int. J. Mol. Sci..

[60] Zhang A., Jung E.-C., Zhu H., Zou Y., Hui X., Maibach H. (2017). Vehicle Effects on Human Stratum Corneum Absorption and Skin Penetration. Toxicol Ind. Health.

[61] Rashid A., White E.T., Howes T., Litster J.D., Marziano I. (2014). Effect of Solvent Composition and Temperature on the Solubility of Ibuprofen in Aqueous Ethanol. J. Chem. Eng. Data.

[62] Watkinson R.M., Herkenne C., Guy R.H., Hadgraft J., Oliveira G., Lane M.E. (2009). Influence of Ethanol on the Solubility, Ionization and Permeation Characteristics of Ibuprofen in Silicone and Human Skin. Skin Pharmacol. Physiol..

[63] Bommannan D., Potts R.O., Guy R.H. (1991). Examination of the Effect of Ethanol on Human Stratum Corneum in Vivo Using Infrared Spectroscopy. J. Control. Release.

[64] Ali A., Garg P., Goyal R., Kaur G., Li X., Negi P., Valis M., Kuca K., Kulshrestha S. (2020). A Novel Herbal Hydrogel Formulation of Moringa Oleifera for Wound Healing. Plants.

[65] Zagórska-Dziok M., Kleczkowska P., Olędzka E., Figat R., Sobczak M. (2021). Poly(Chitosan-Ester-Ether-Urethane) Hydrogels as Highly Controlled Genistein Release Systems. IJMS.

[66] Mandal A., Clegg J.R., Anselmo A.C., Mitragotri S. (2020). Hydrogels in the Clinic. Bioeng. Transl. Med..

[67] Champeau M., Seabra A.B., de Oliveira M.G. (2017). Hydrogels for Topical Nitric Oxide Delivery. Nitric Oxide Donors.

[68] Ossowicz P., Klebeko J., Janus E., Nowak A., Duchnik W., Kucharski Ł., Klimowicz A. (2020). The Effect of Alcohols as Vehicles on the Percutaneous Absorption and Skin Retention of Ibuprofen Modified with l-Valine Alkyl Esters. RSC Adv..

[69] Harwansh R.K., Mukherjee P.K., Bahadur S., Biswas R. (2015). Enhanced Permeability of Ferulic Acid Loaded Nanoemulsion Based Gel through Skin against UVA Mediated Oxidative Stress. Life Sci..

[70] Campanini M.Z., Pinho-Ribeiro F.A., Ivan A.L.M., Ferreira V.S., Vilela F.M.P., Vicentini F.T.M.C., Martinez R.M., Zarpelon A.C., Fonseca M.J.V., Faria T.J. (2013). Efficacy of Topical Formulations Containing Pimenta Pseudocaryophyllus Extract against UVB-Induced Oxidative Stress and Inflammation in Hairless Mice. J. Photochem. Photobiol. B Biol..

[71] Borges A., Serra S., Cristina Abreu A., Saavedra M.J., Salgado A., Simões M. (2014). Evaluation of the Effects of Selected Phytochemicals on Quorum Sensing Inhibition and in Vitro Cytotoxicity. Biofouling.

[72] Truzzi F., Valerii M.C., Tibaldi C., Zhang Y., Abduazizova V., Spisni E., Dinelli G. (2020). Are Supplements Safe? Effects of Gallic and Ferulic Acids on In Vitro Cell Models. Nutrients.

[73] Choi J.-H., Park J.-K., Kim K.-M., Lee H.-J., Kim S. (2018). In Vitro and in Vivo Antithrombotic and Cytotoxicity Effects of Ferulic Acid. J. Biochem. Mol. Toxicol..

[74] Wang T., Gong X., Jiang R., Li H., Du W., Kuang G. (2016). Ferulic Acid Inhibits Proliferation and Promotes Apoptosis via Blockage of PI3K/Akt Pathway in Osteosarcoma Cell. Am. J. Transl. Res..

[75] Gao J., Yu H., Guo W., Kong Y., Gu L., Li Q., Yang S., Zhang Y., Wang Y. (2018). The Anticancer Effects of Ferulic Acid Is Associated with Induction of Cell Cycle Arrest and Autophagy in Cervical Cancer Cells. Cancer Cell. Int..

[76] Zhang X., Lin D., Jiang R., Li H., Wan J., Li H. (2016). Ferulic Acid Exerts Antitumor Activity and Inhibits Metastasis in Breast Cancer Cells by Regulating Epithelial to Mesenchymal Transition. Oncol. Rep..

