# Supplementary material for: New Ferulic Acid and Amino Acid Derivatives with Increased Cosmeceutical and Pharmaceutical Potential
Source: Pharmaceutics. 2022 Dec 29;15(1):117. doi: 10.3390/pharmaceutics15010117 (PMC9861623; doi:10.3390/pharmaceutics15010117)

pharmaceutics

Supplementary Material

New Ferulic Acid and Amino Acid Derivatives with Increased Cosmeceutical and Pharmaceutical Potential

Ewa Janus [1,*], Luan Ramalho Pinheiro [1,2], Anna Nowak [3], Edyta Kucharska [1], Ewelina Świątek [1], Natalia Podolak [1], Magdalena Perużyńska [4], Katarzyna Piotrowska [5], Wiktoria Duchnik [3], Łukasz Kucharski [3] and Adam Klimowicz [3,*]

[1] Department of Chemical Organic Technology and Polymeric Materials, Faculty of Chemical Technology and Engineering, West Pomeranian University of Technology in Szczecin, Pulaski Ave. 10, 70-322 Szczecin, Poland
[2] Polytechnic Institute of PUC Minas, Pontifical Catholic University of Minas Gerais, Belo Horizonte 30535-901, Brazil
[3] Department of Cosmetic and Pharmaceutical Chemistry, Pomeranian Medical University in Szczecin, 70-111 Szczecin, Poland
[4] Department of Experimental & Clinical Pharmacology, Pomeranian Medical University in Szczecin, 70-111 Szczecin, Poland
[5] Department of Physiology, Pomeranian Medical University in Szczecin, 70-111 Szczecin, Poland
* Correspondence: ejanus@zut.edu.pl (E.J.); adam.klimowicz@pum.edu.pl (A.K.)

Table of contents:

S1. **Copies of ^{1}H and ^{13}C NMR of ferulic acid** **page 1**
S2. **Copies of ^{1}H NMR, ^{13}C NMR and ^{1}H-^{13}C HMQC spectra of amino acid propyl esters ferulates** **pages 2–6**
S3. **FTIR spectra of ferulic acid and amino acid propyl esters ferulates** **pages 6–9**
S4. **DSC curves for ferulic acid and amino acid propyl esters ferulates** **pages 10–11**
S5. **Curves from thermogravimetric analysis of ferulic acid and amino acid propyl esters ferulates** **pages 12–13**
S6. **Photographs of hydrogels and emulsions preparations** **pages 14–15**

S1. Copies of ^{1}H and ^{13}C NMR spectra of ferulic acid

^{1}H NMR spectrum of ferulic acid (400 MHz, DMSO-d_6)

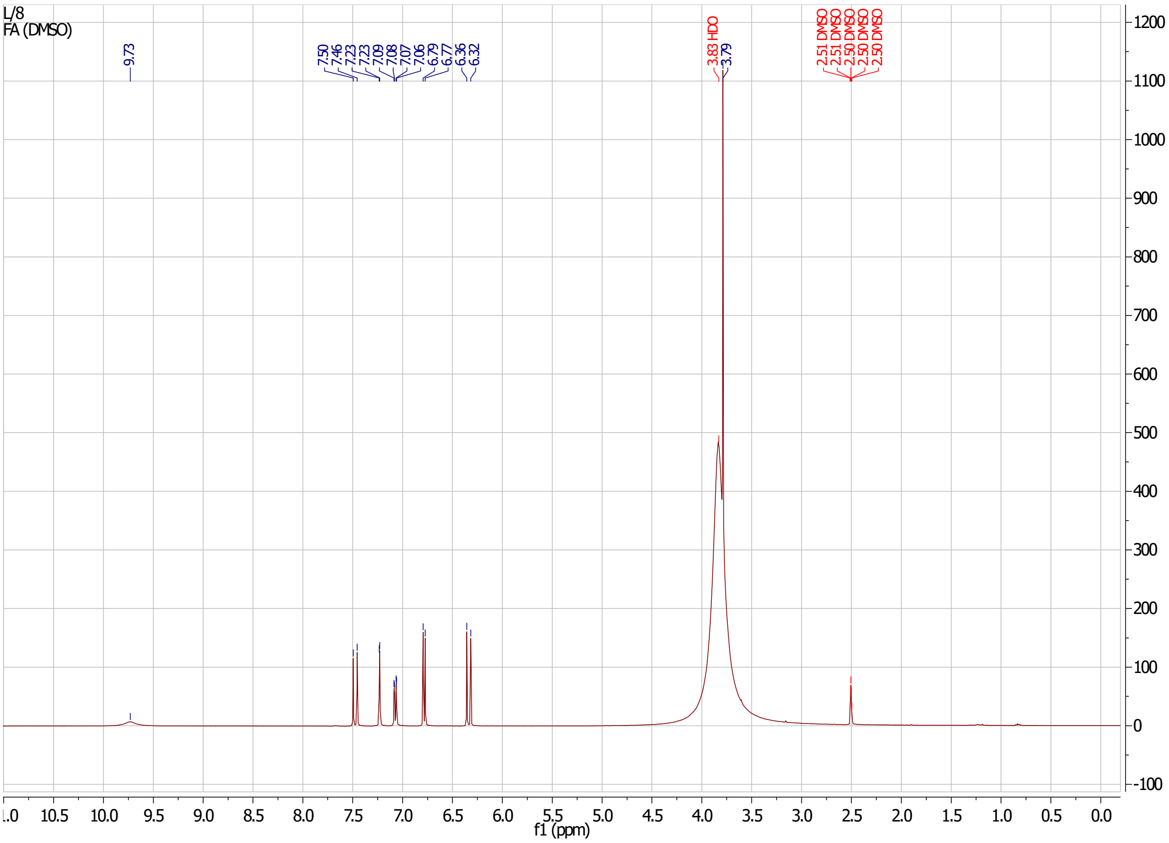

^{13}C NMR spectrum of ferulic acid (101 MHz, DMSO-d_6)

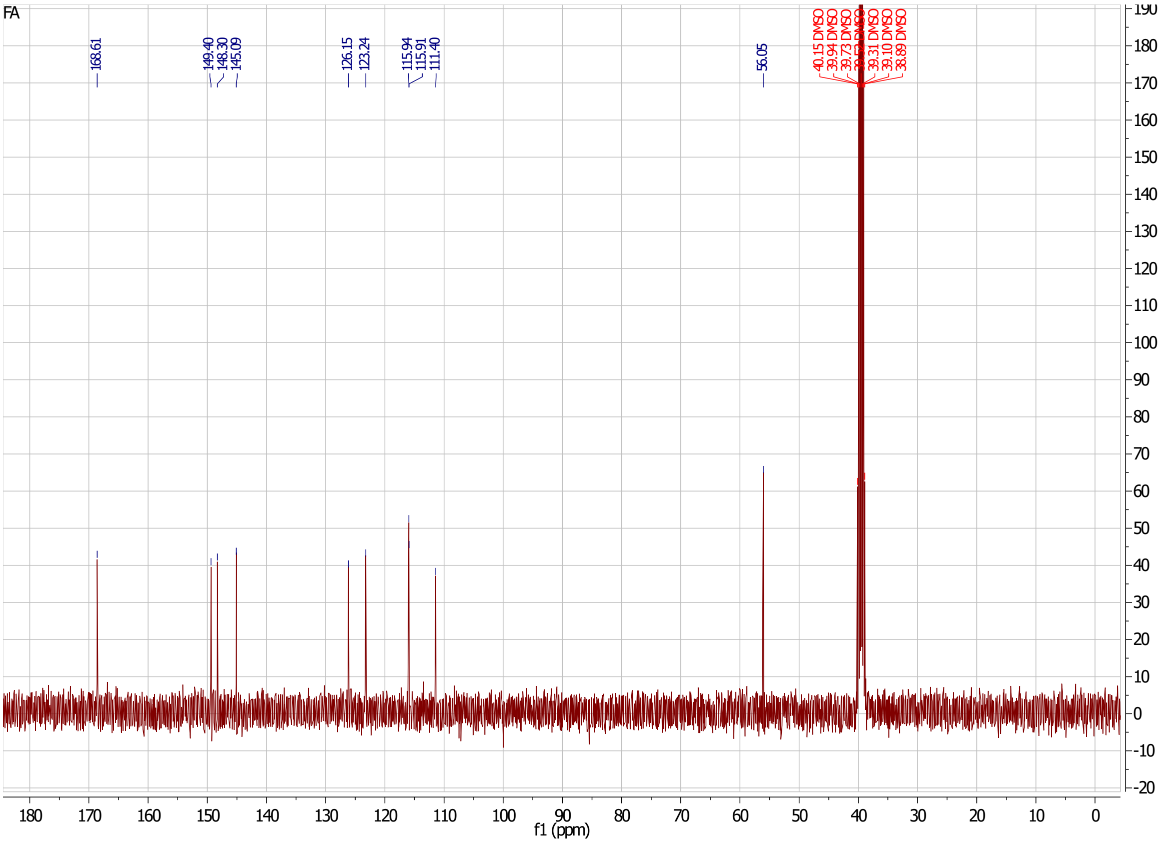

S2. Copies of ^{1}H NMR, ^{13}C NMR and HMQC spectra of amino acid propyl esters ferulates

^{1}H NMR spectrum of glycine propyl ester ferulate (400 MHz, DMSO-*d*6)

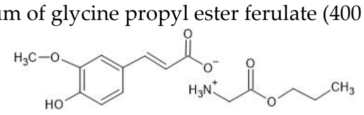

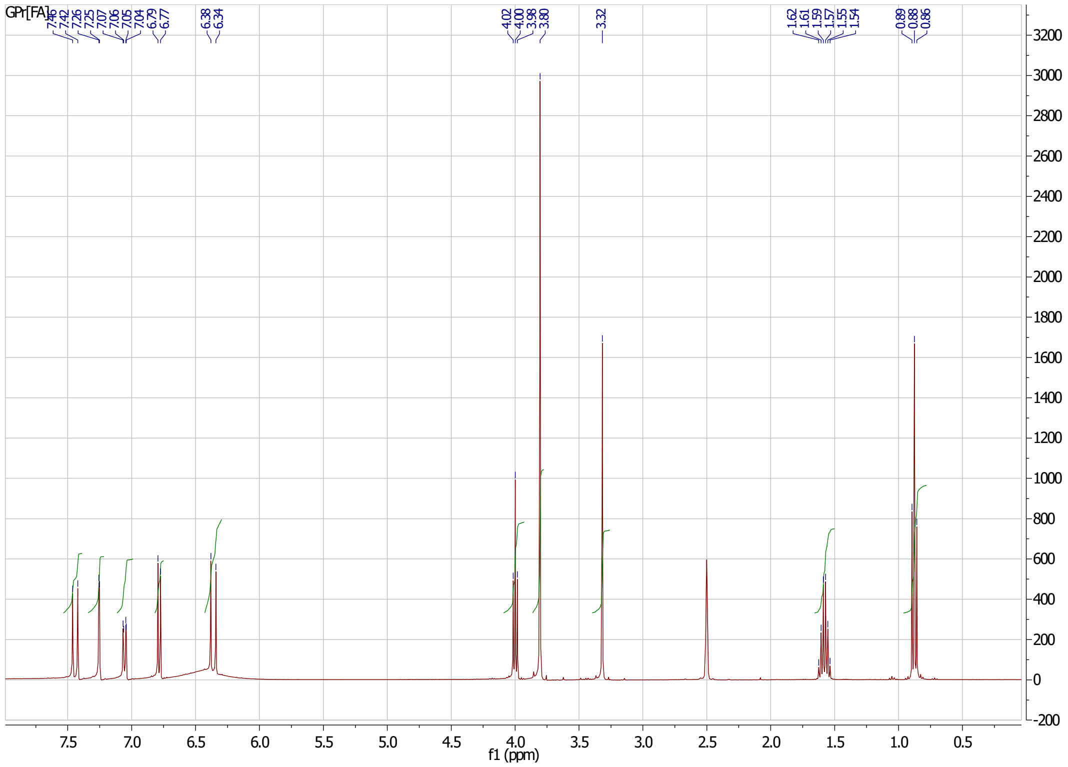

^{13}C NMR spectrum of glycine propyl ester ferulate (101 MHz, DMSO-*d*6)

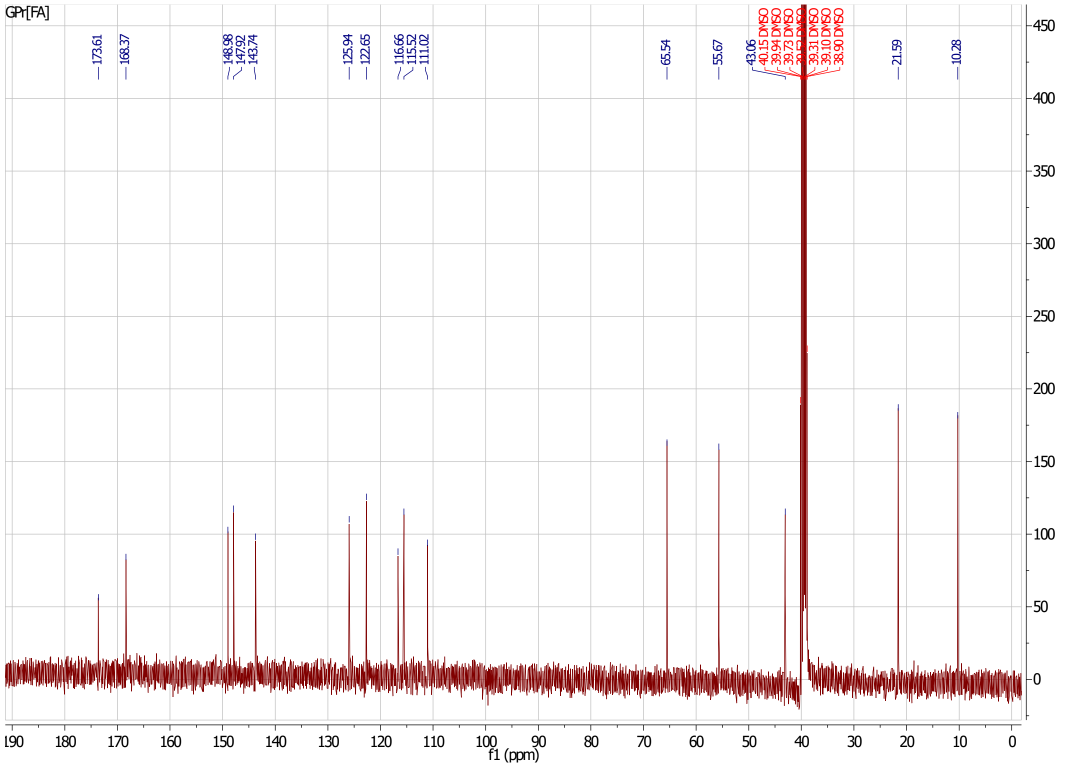

^{1}H-^{13}C HMQC spectrum of glycine propyl ester ferulate

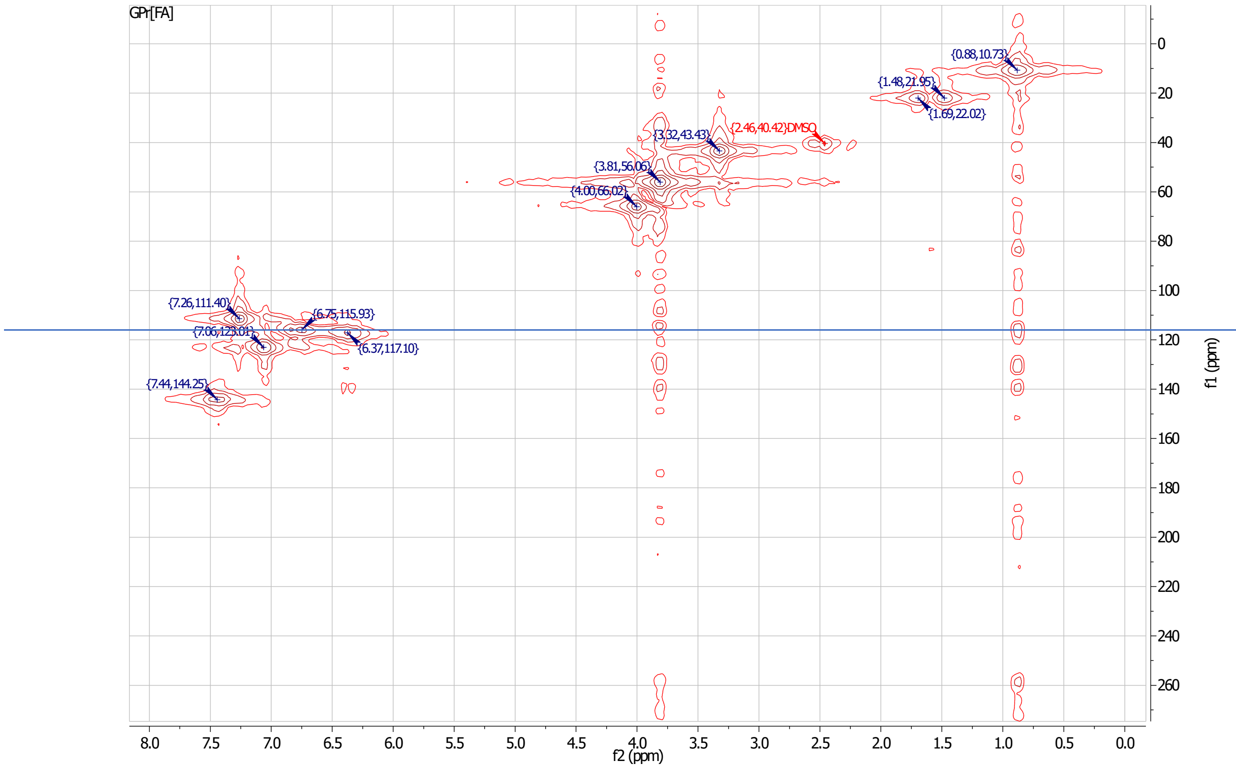

^{1}H NMR spectrum of L-leucine propyl ester ferulate (400 MHz, DMSO-d_6)

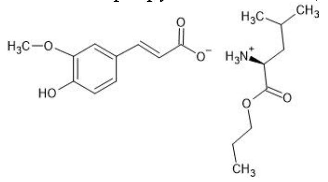

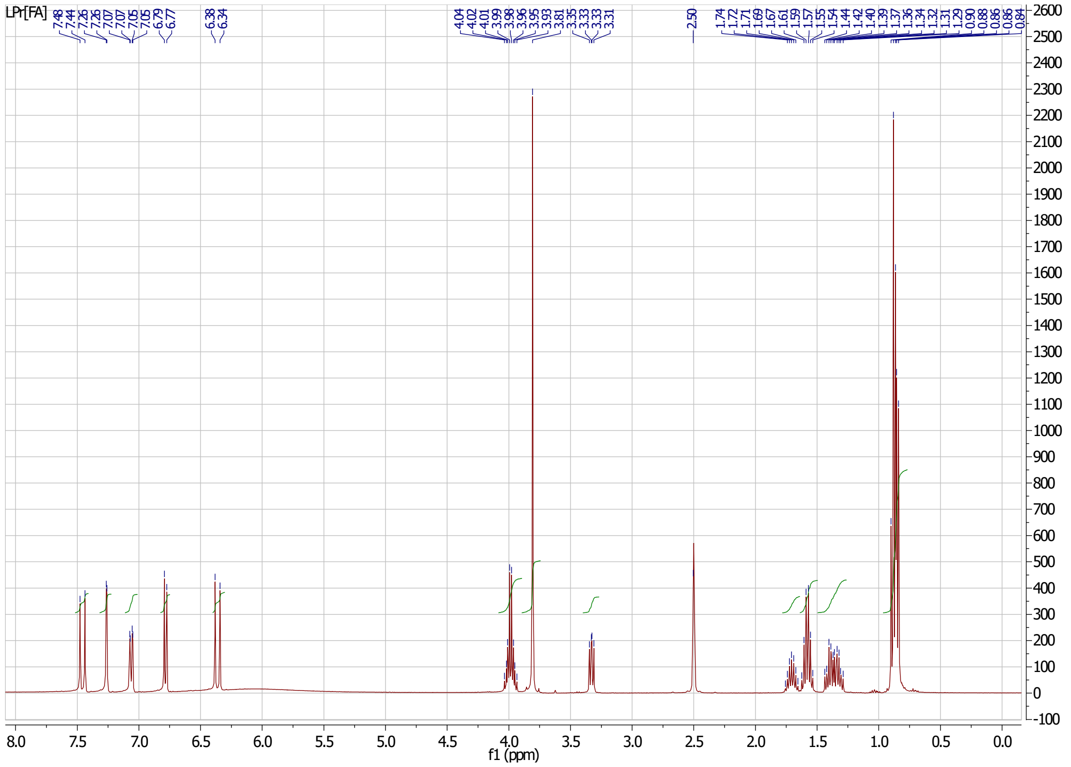

^{13}C NMR spectrum of L-leucine propyl ester ferulate (101 MHz, DMSO-d_6)

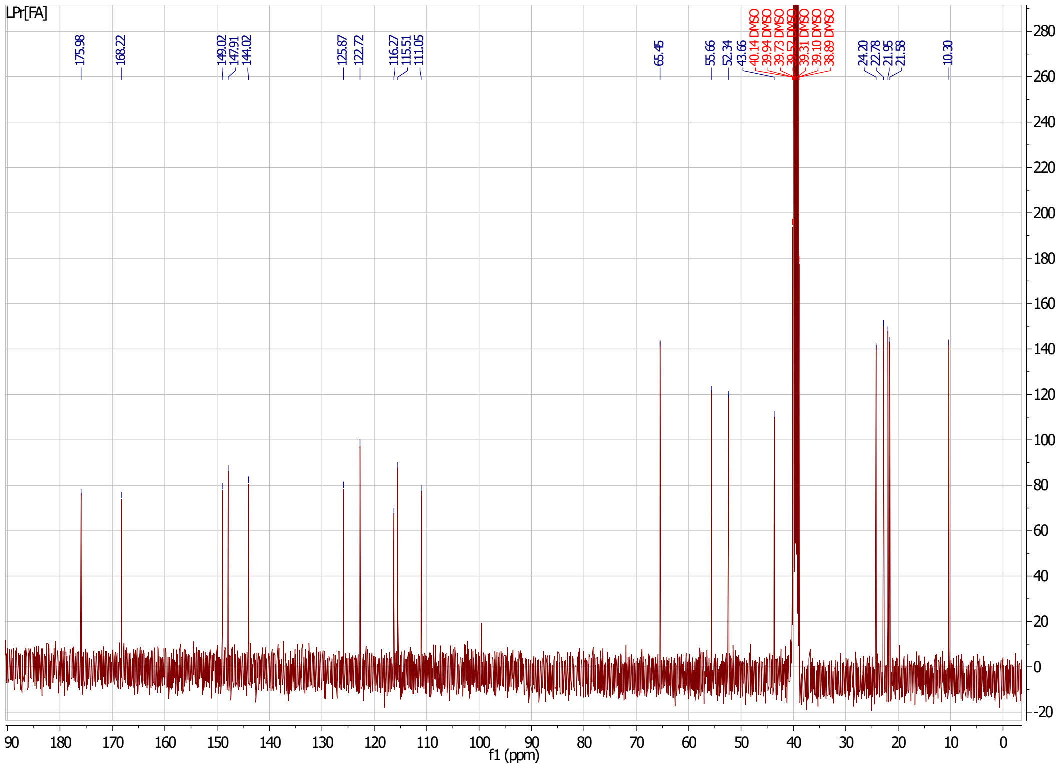

^{1}H-^{13}C HMQC spectrum of L-leucine propyl ester ferulate

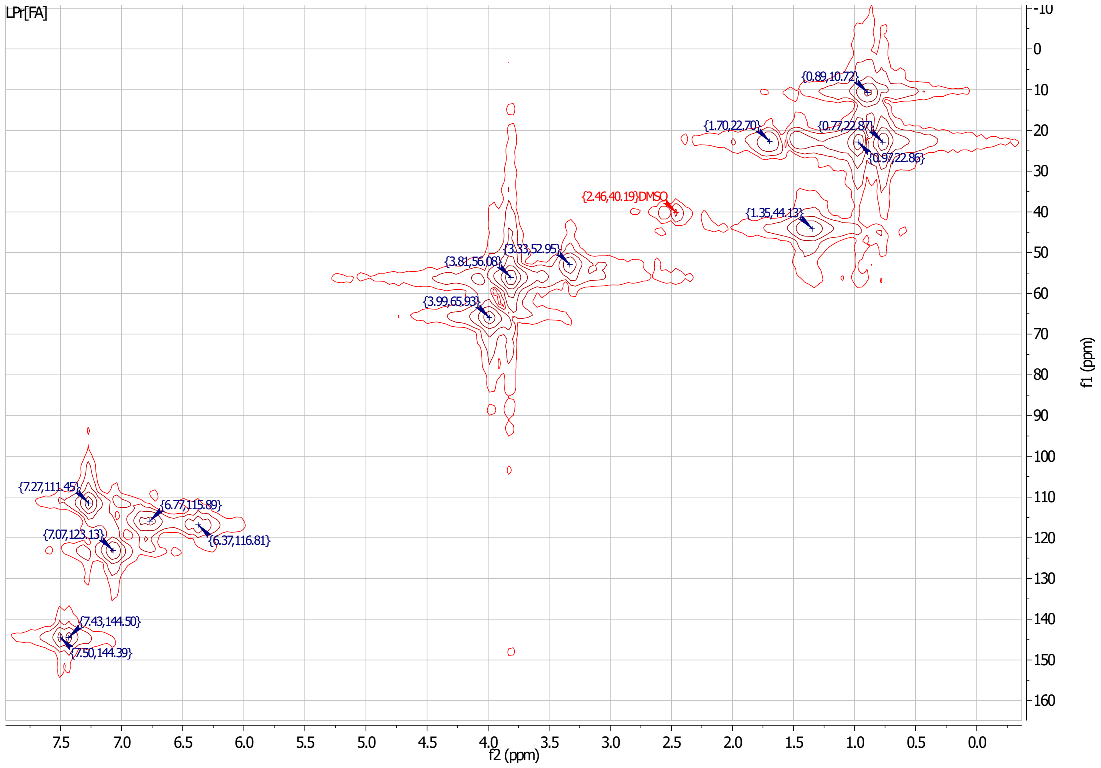

^{1}H NMR spectrum of L-proline propyl ester ferulate (400 MHz, DMSO-d_6)

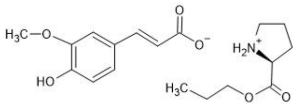

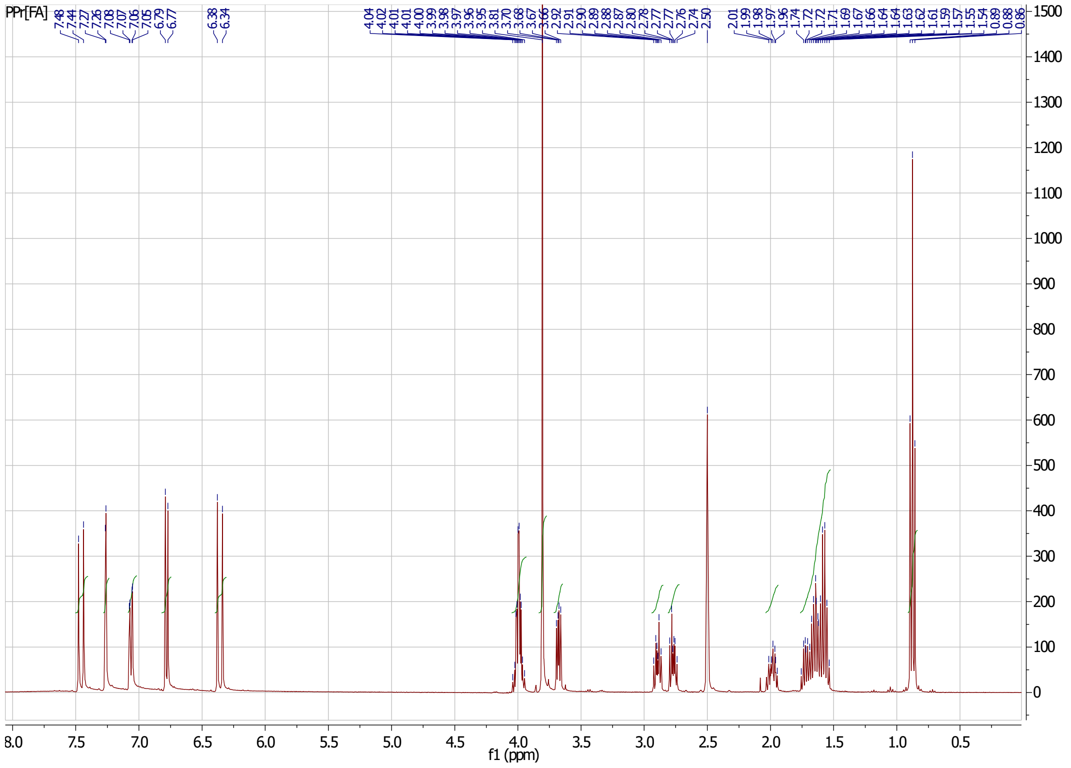

^{13}C NMR spectrum of L-proline propyl ester ferulate (101 MHz, DMSO-*d*$_6$)

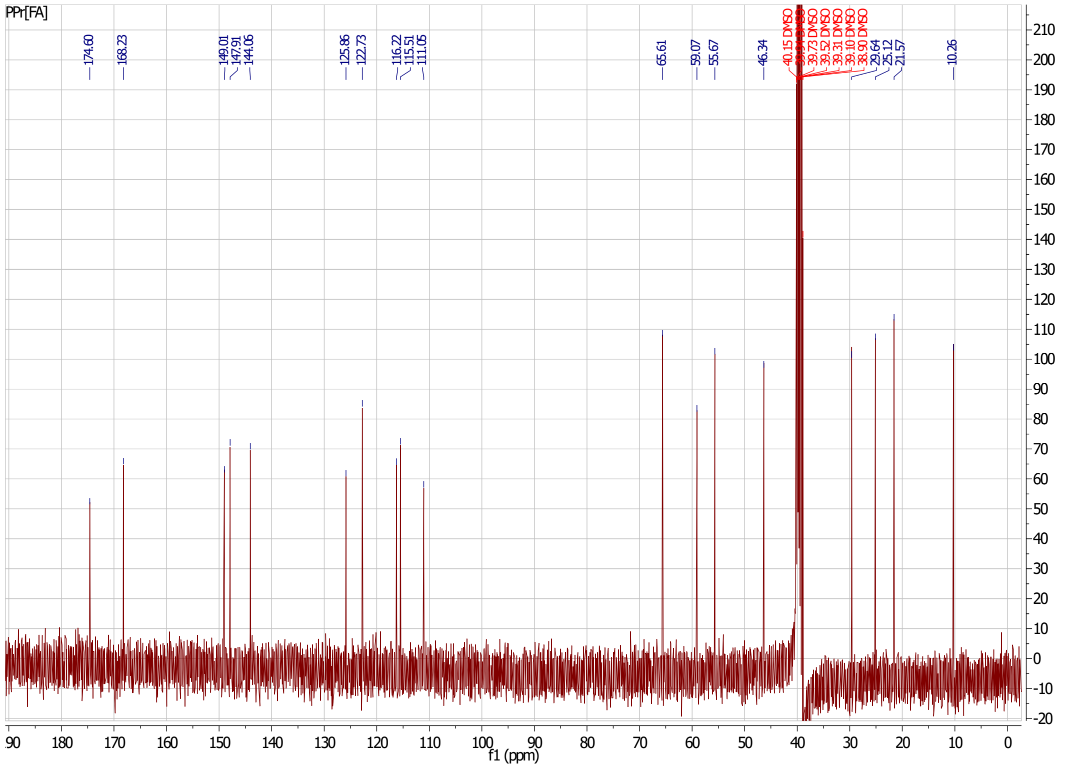

^{1}H-^{13}C HMQC spectrum of L-proline propyl ester ferulate

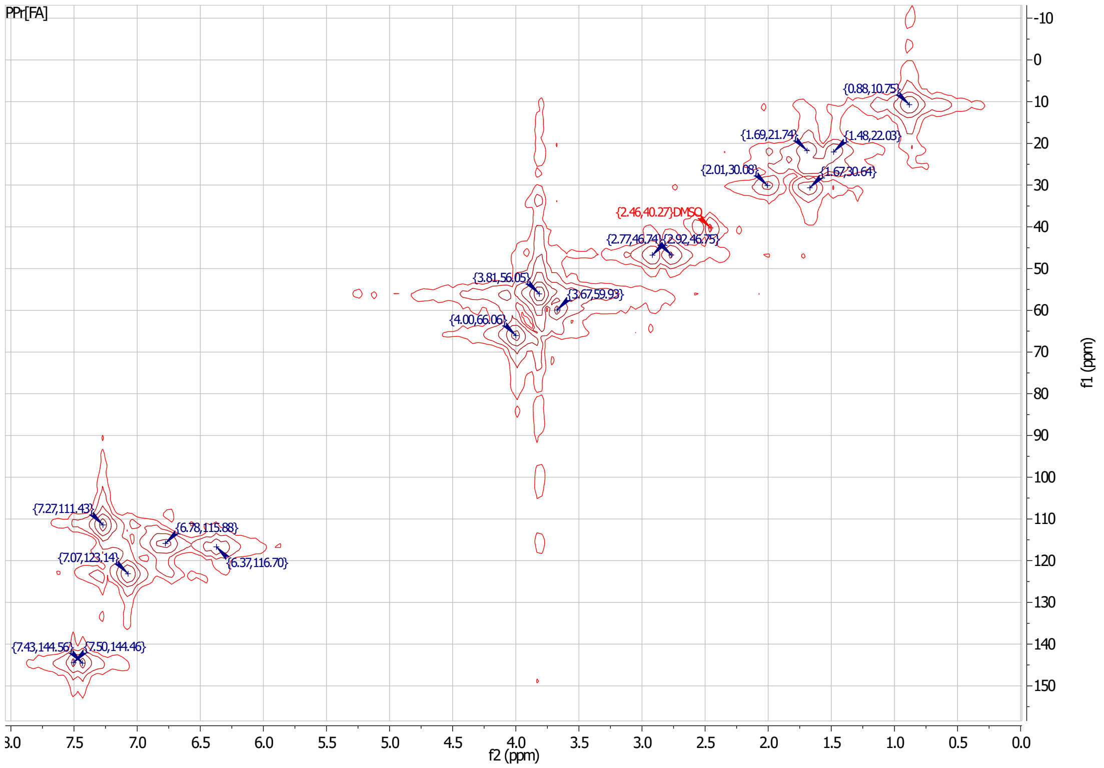

S3. FTIR spectra of ferulic acid and amino acid propyl esters ferulates

FTIR spectrum of ferulic acid

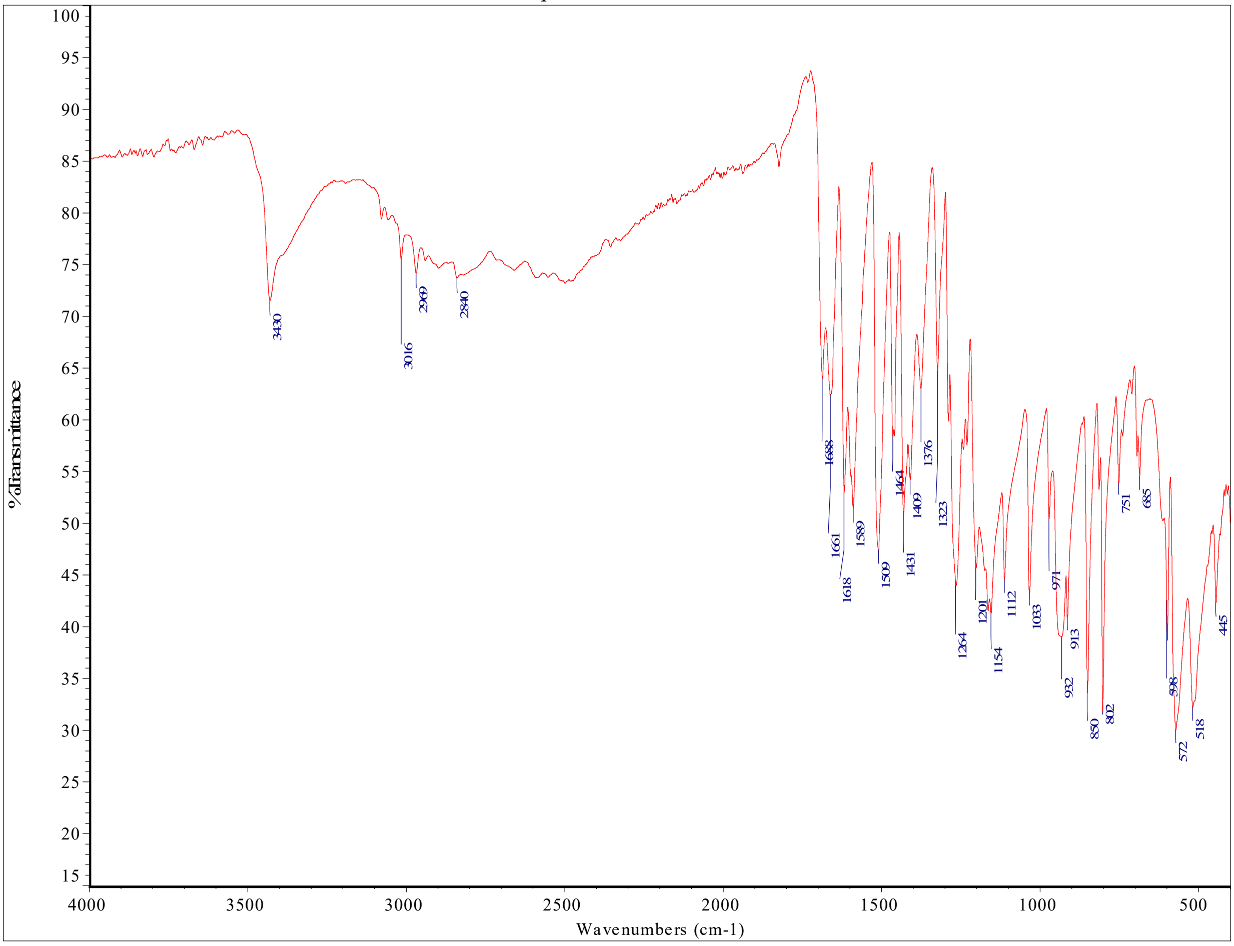

FTIR spectrum of glycine propyl ester ferulate, GPr[FA]

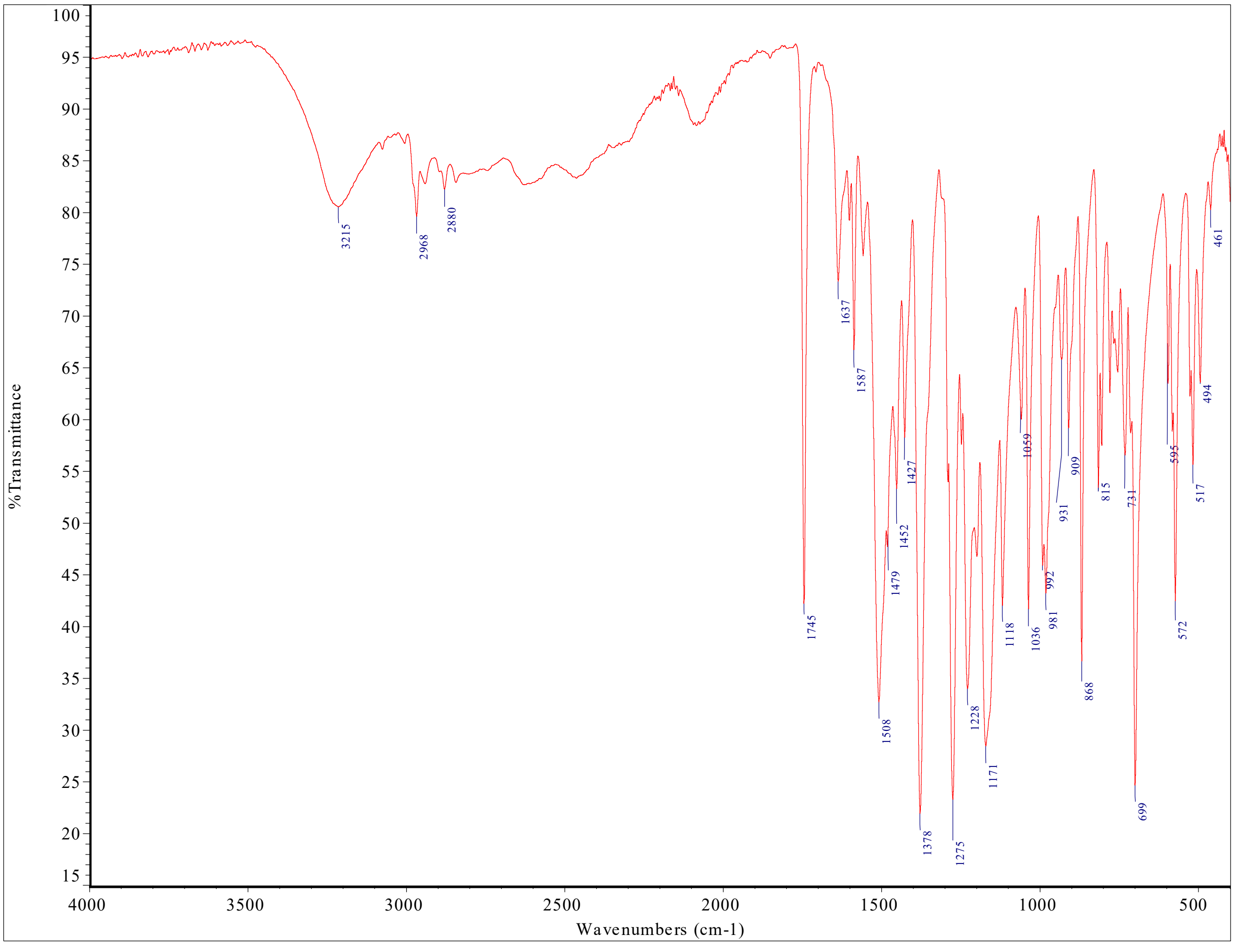

FTIR spectrum of L-leucine propyl ester ferulate, LPr[FA]

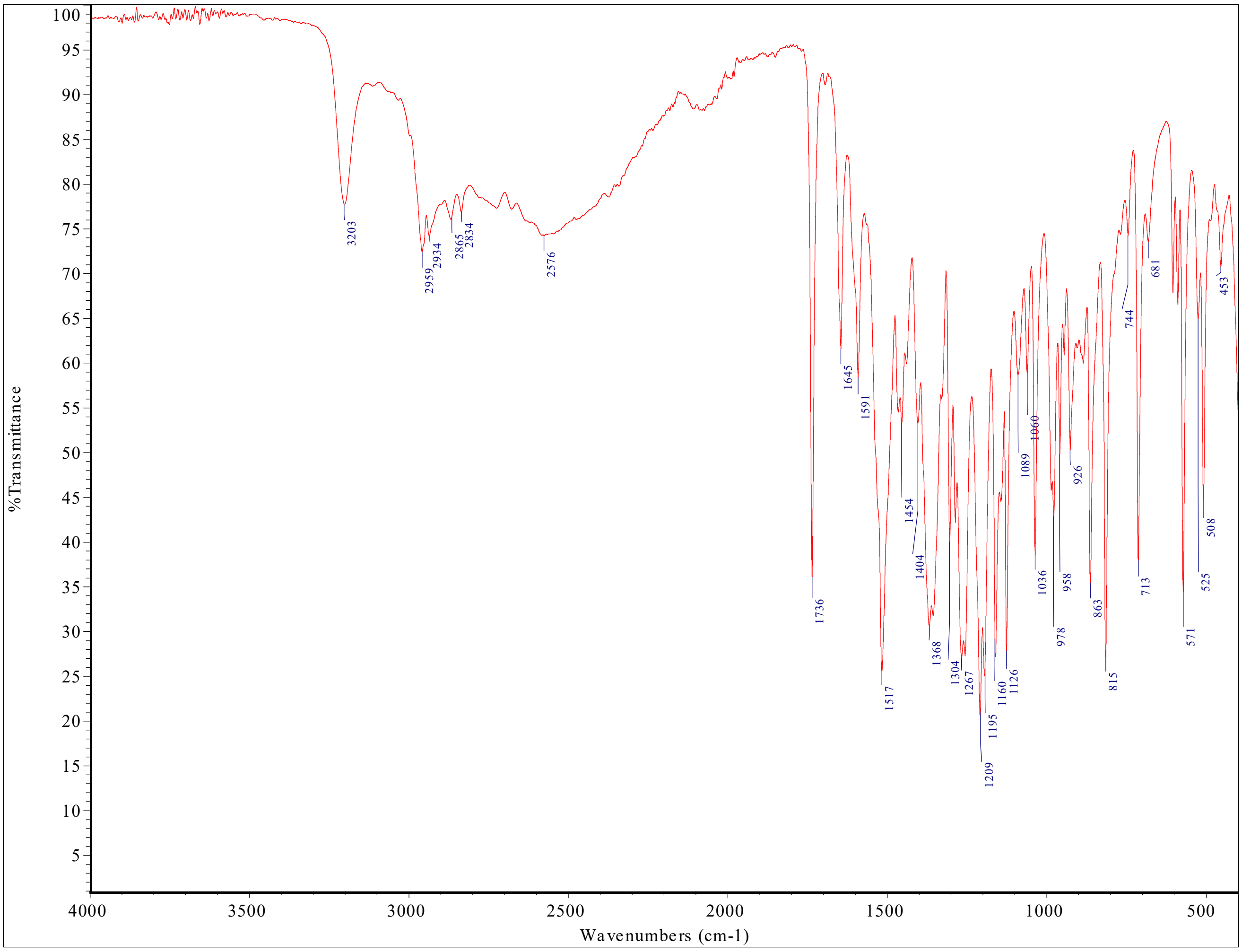

FTIR spectrum of L-proline propyl ester ferulate, PPr[FA]

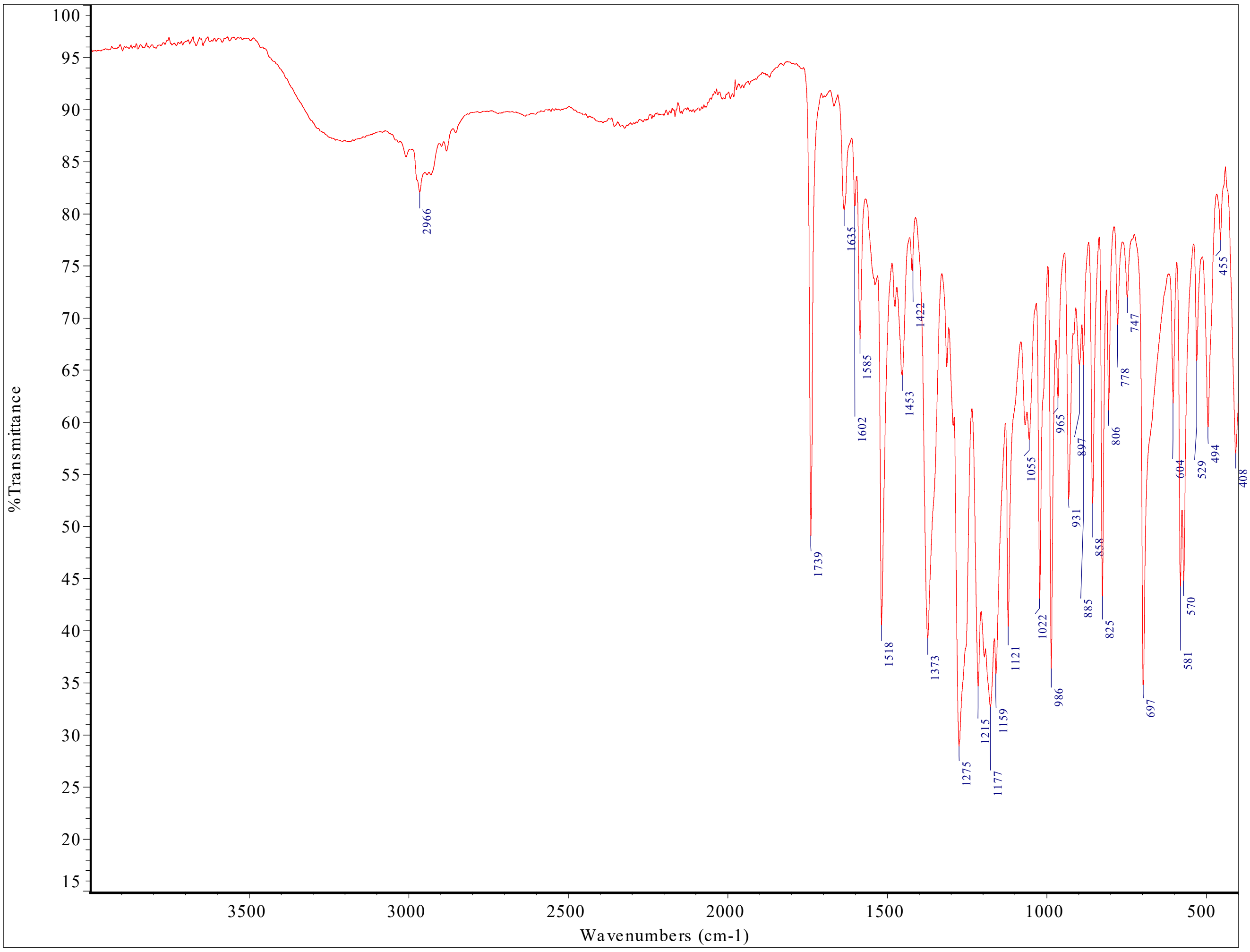

S4. DSC curves for ferulic acid and amino acid propyl esters ferulates

DSC curves for ferulic acid (FA) on heating-cooling-heating regime in the range of temperature 20ºC – 210ºC

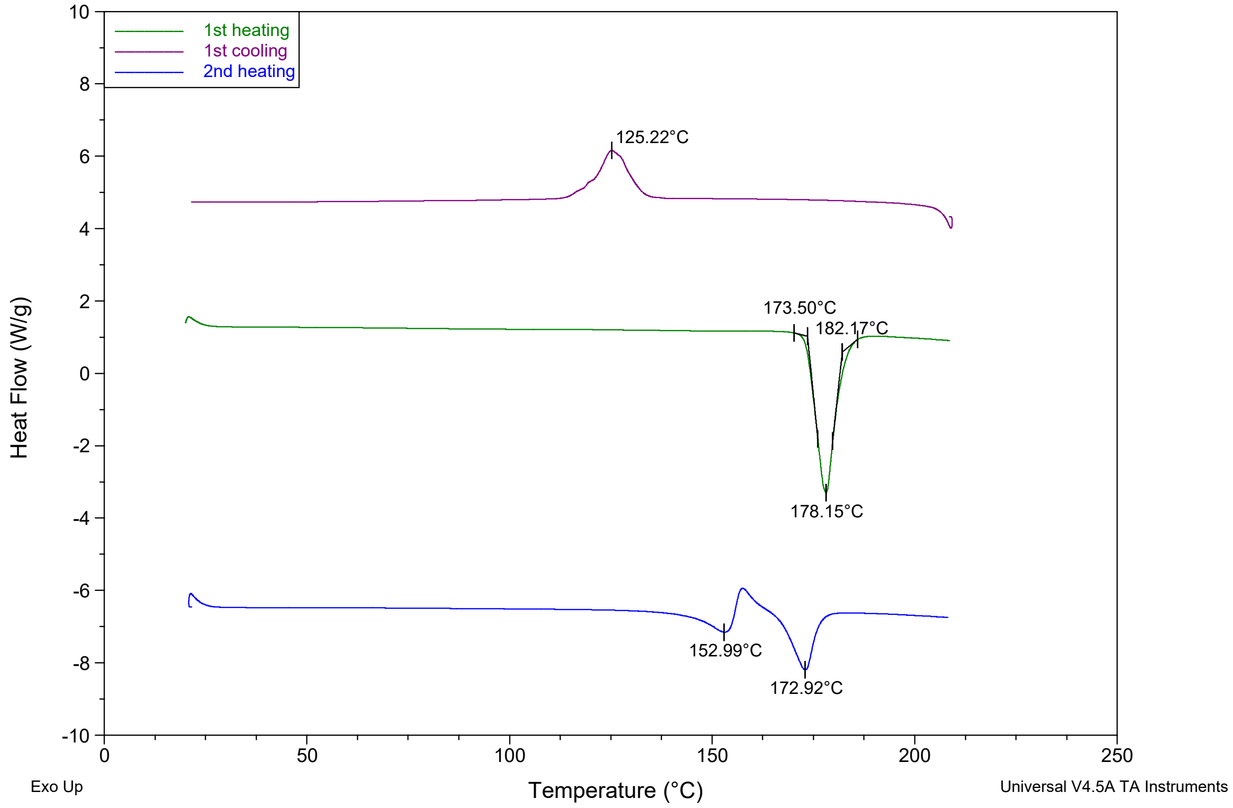

DSC curves for glycine propyl ester ferulate, GPr[FA] on heating-cooling-heating regime in the range of temperature 20ºC – 170ºC

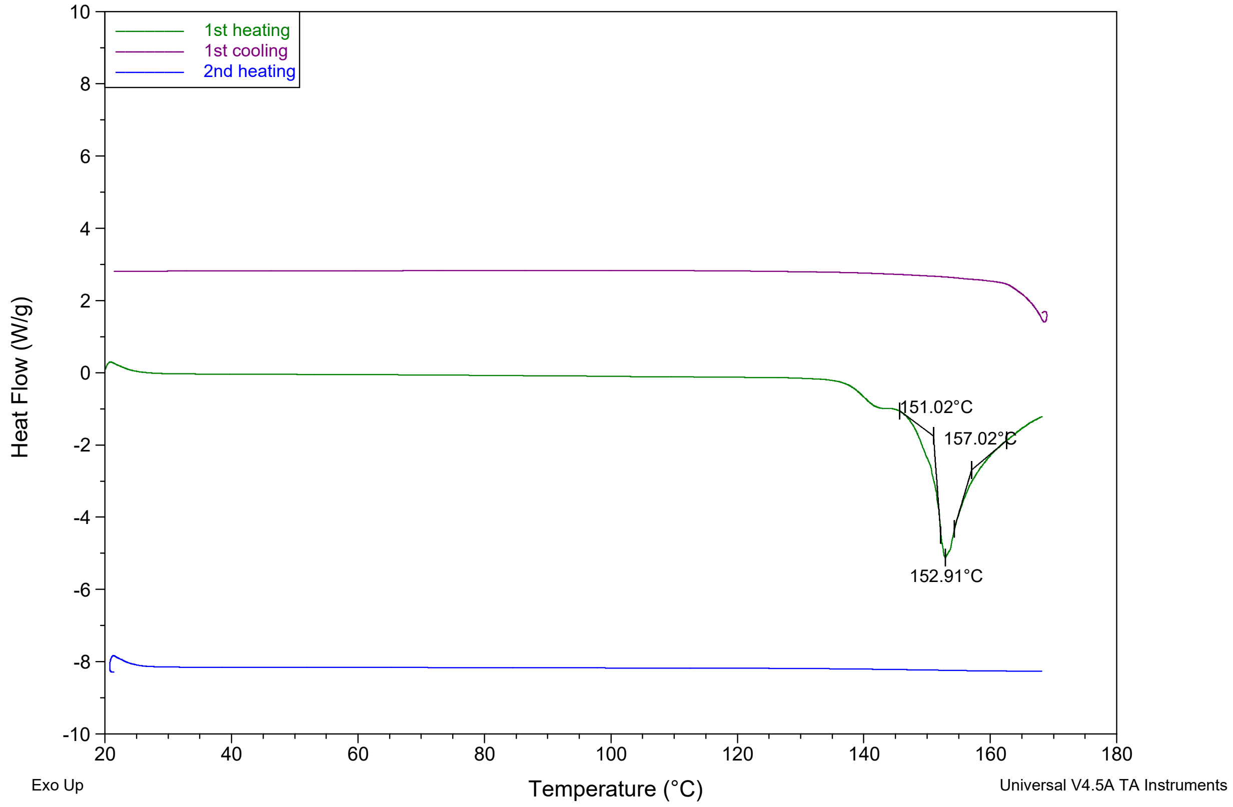

DSC curves for L-leucine propyl ester ferulate, LPr[FA] on heating-cooling-heating regime in the range of temperature 20ºC – 140ºC

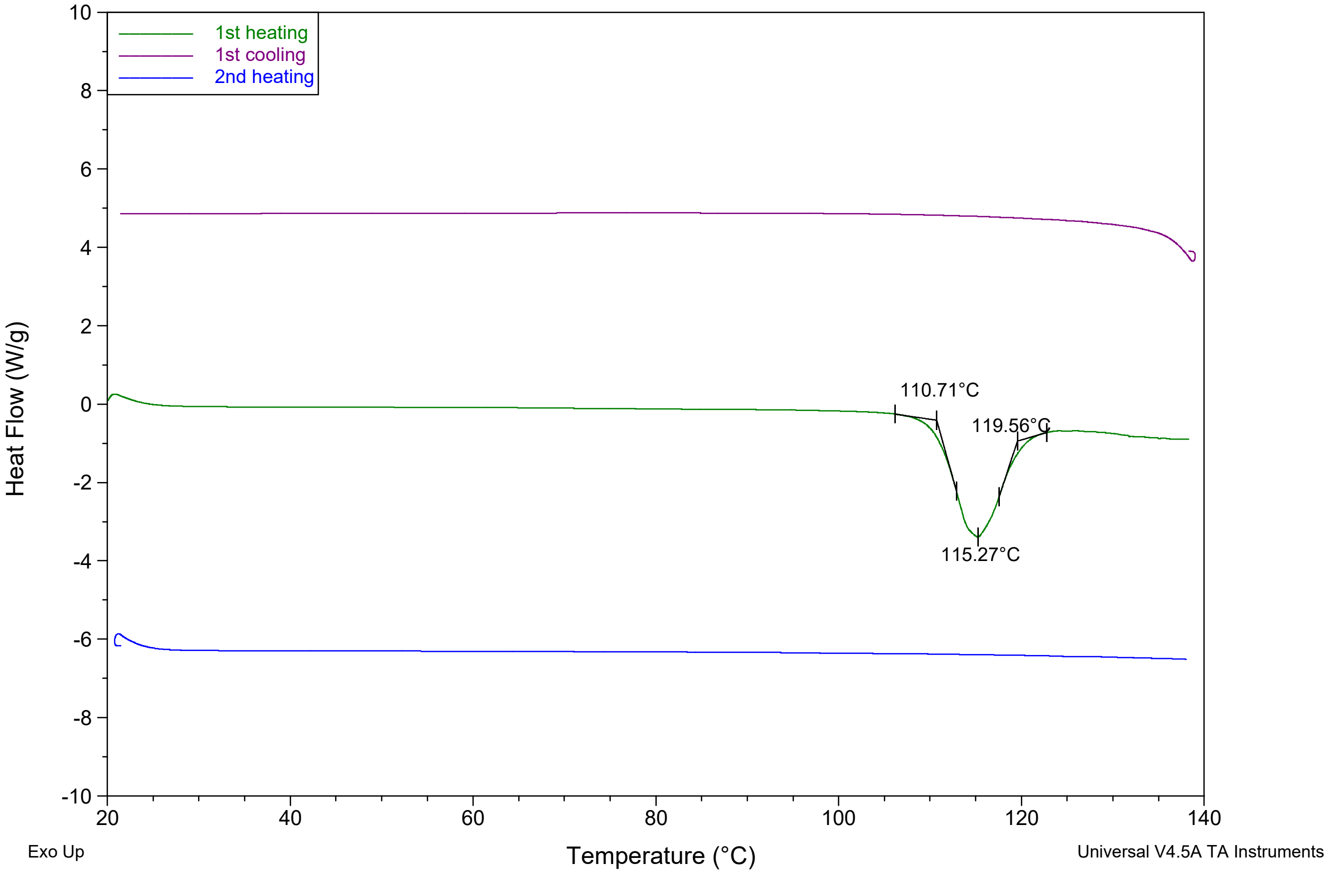

DSC curves for L-proline propyl ester ferulate, PPr[FA] on heating-cooling-heating regime in the range of temperature 20°C – 130°C

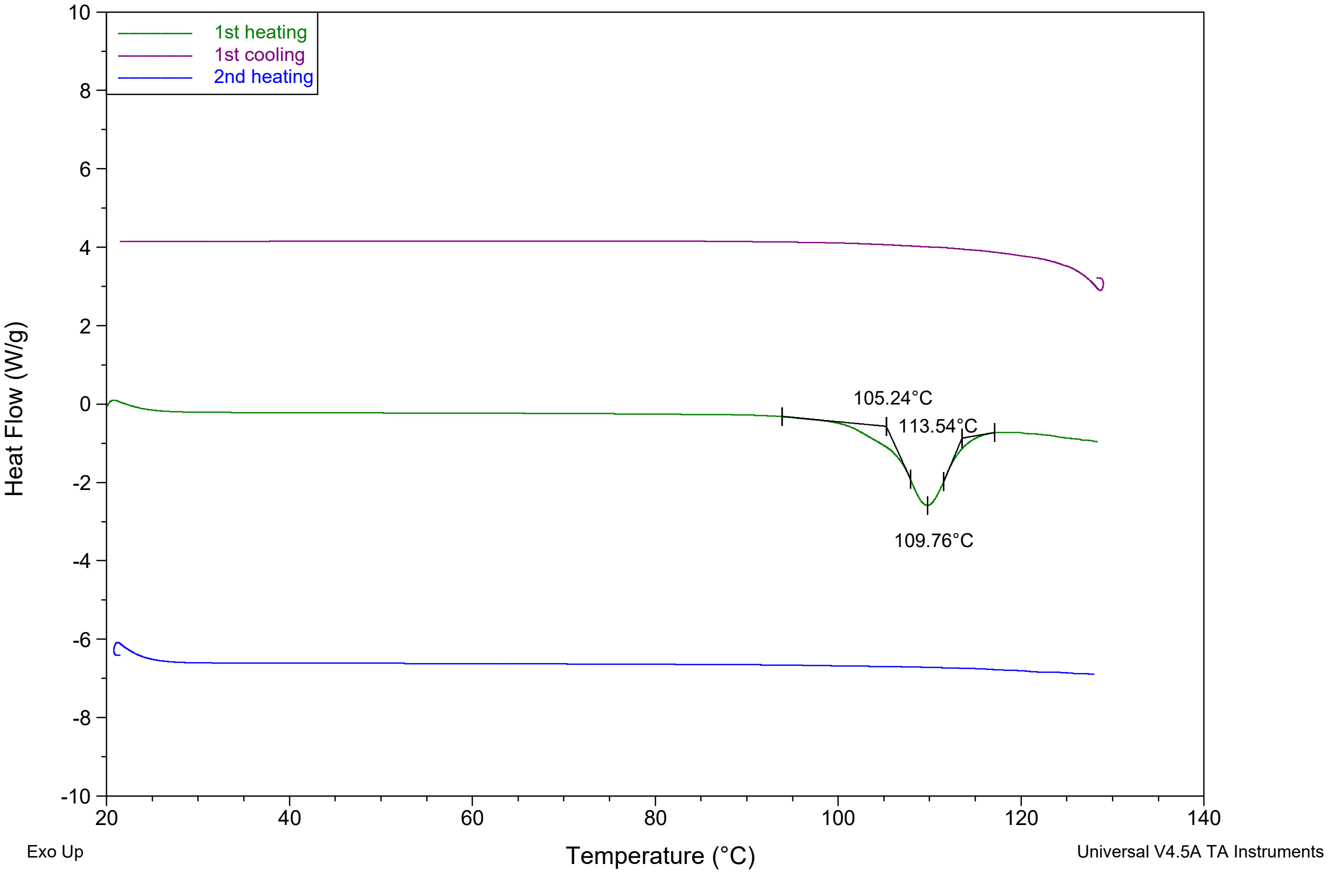

S5. Curves from thermogravimetric analysis of ferulic acid and amino acid propyl esters ferulates

TG, DTG and c-DTA curves for ferulic acid

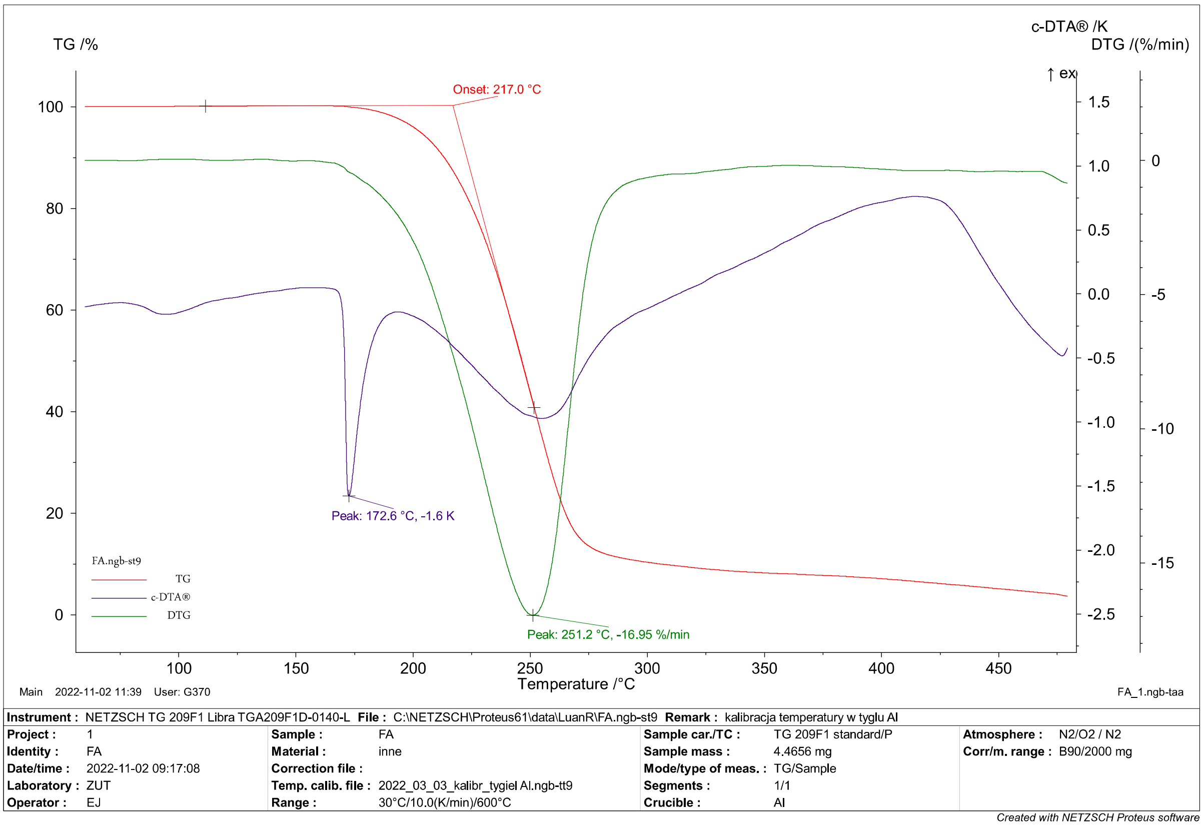

TG, DTG and c-DTA curves for glycine propyl ester ferulate

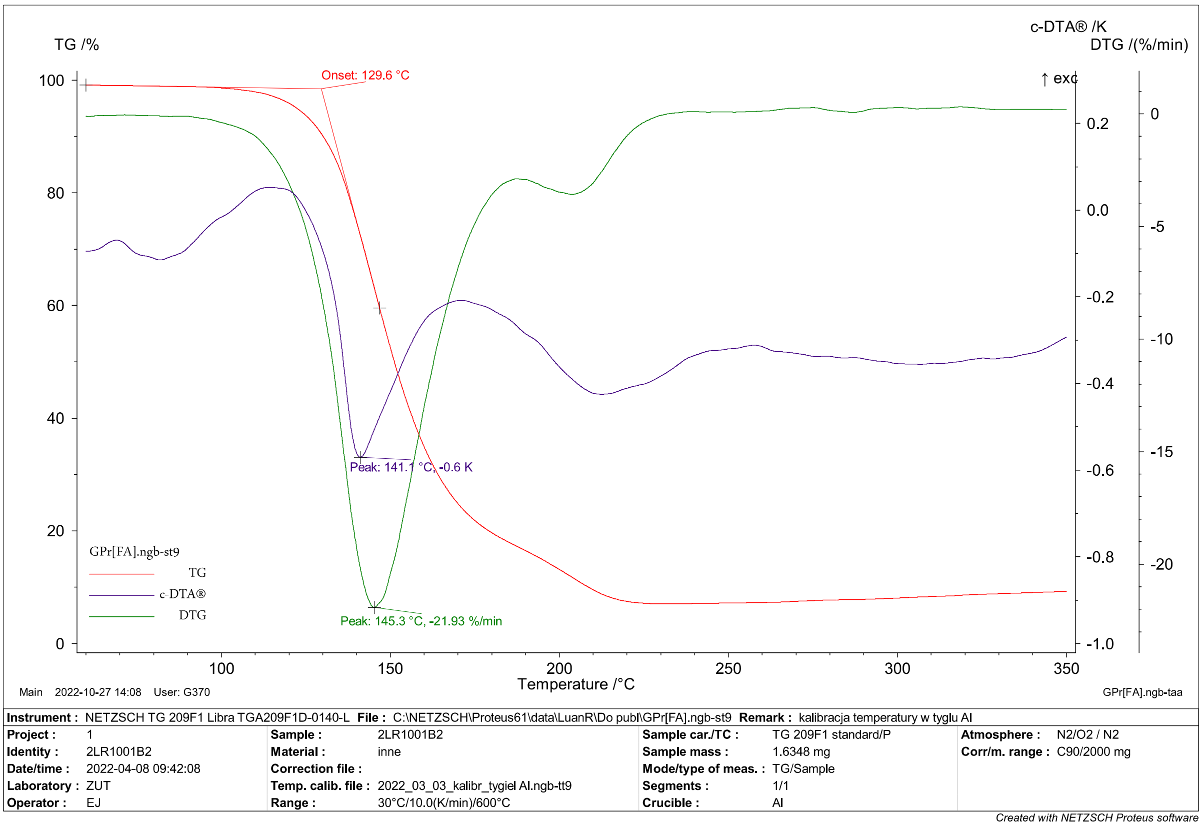

TG, DTG and c-DTA curves for L-leucine propyl ester ferulate

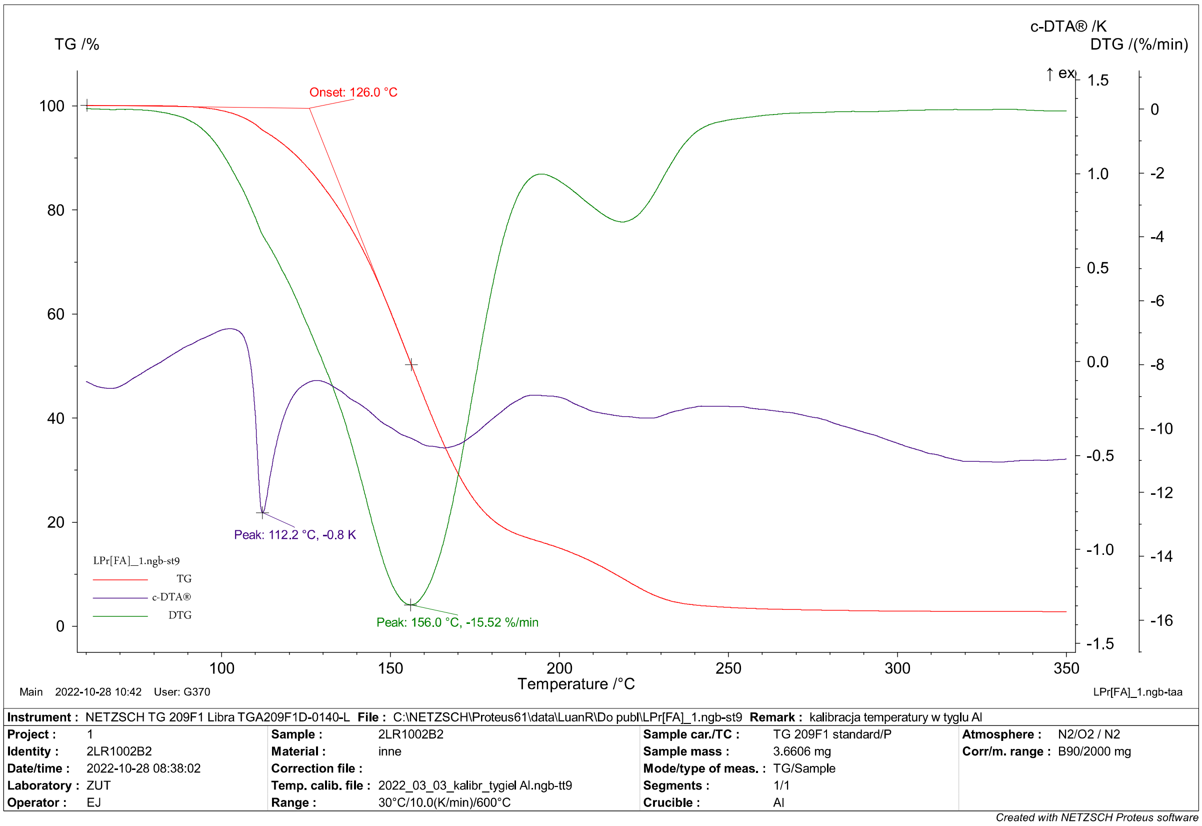

TG, DTG and c-DTA curves for L-proline propyl ester ferulate

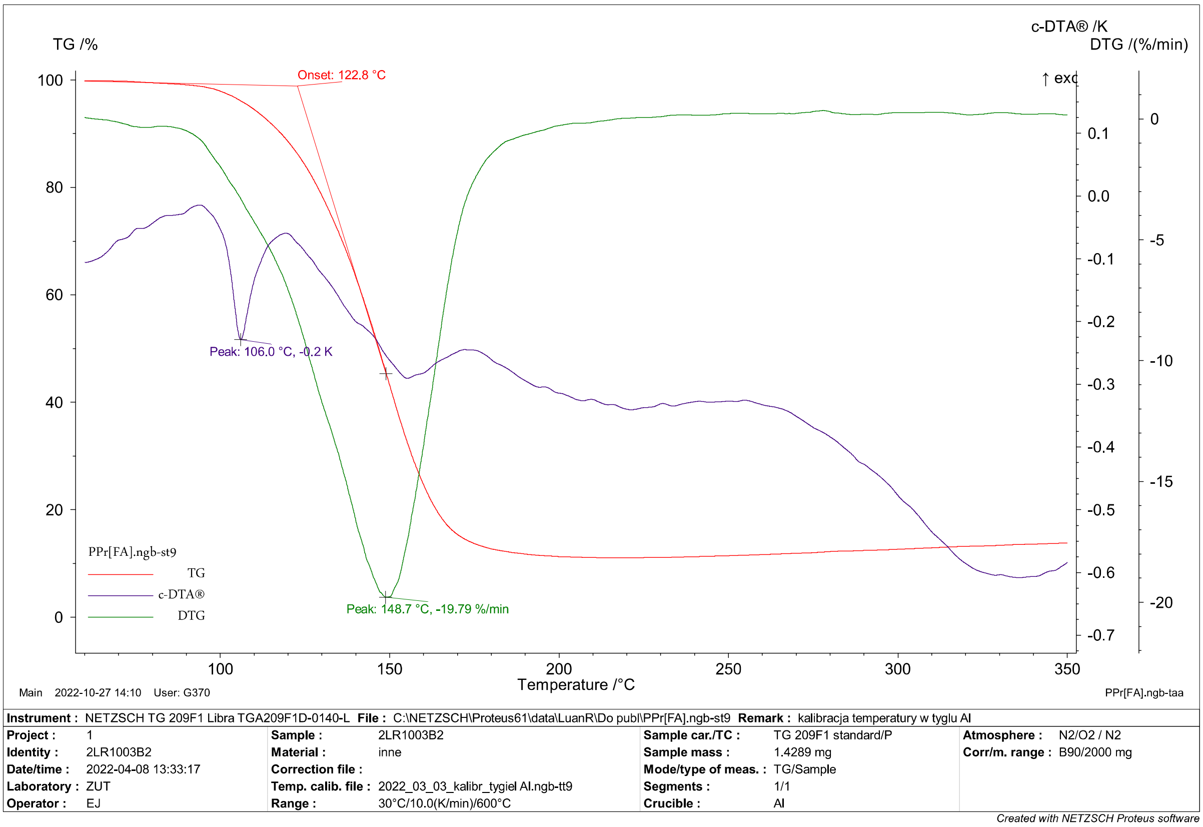

S6. Photographs of hydrogels and emulsions preparations

Emulsions and hydrogels used in the permeation test.

A: the emulsions: 0 – emulsion vehicle (without FA and its derivatives), 1 - E-FA; 2 - GPr[FA], 3 - LPr[FA] and 4 - PPr[FA];
B: the hydrogels: 0 – hydrogel vehicle (without FA and its derivatives), 1 - H-FA; 2-GPr[FA], 3 - LPr[FA] and 4 - PPr[FA].

The photographs of the preparations were done prior applied to skin in the Franz chamber.

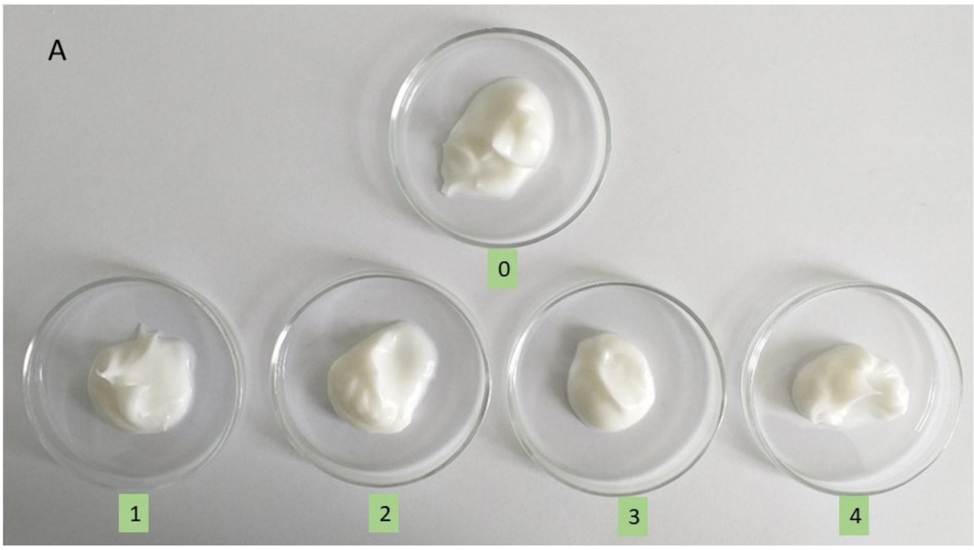

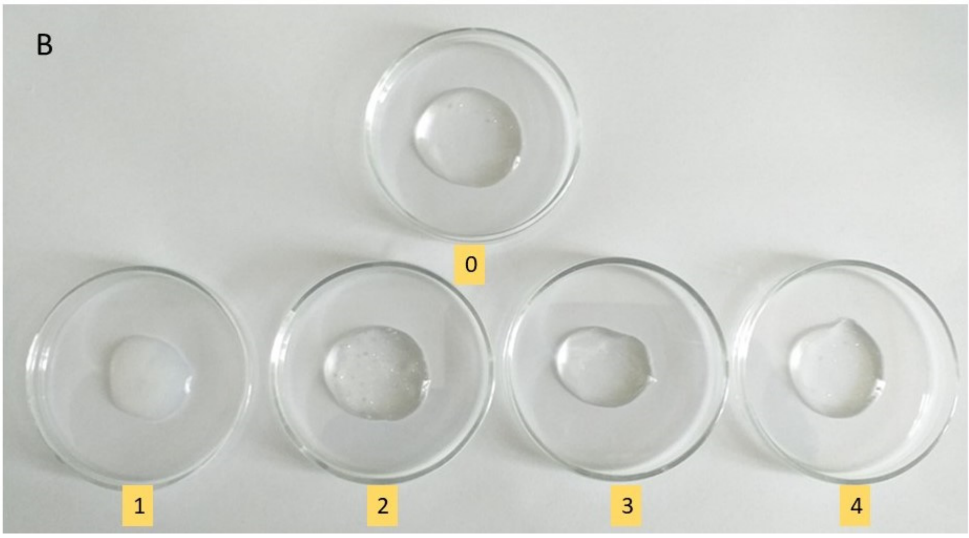

Emulsions and Hydrogels after the stability test

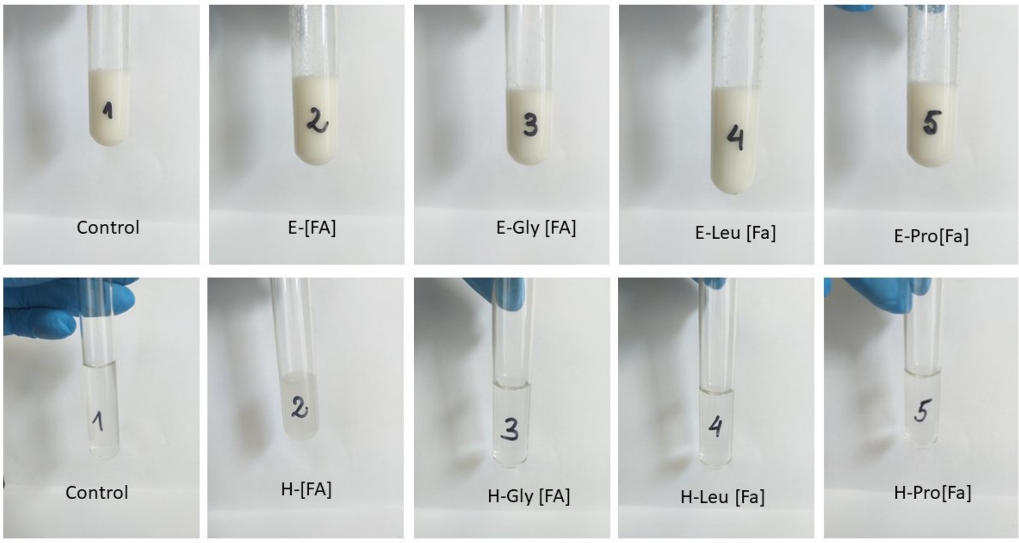

Supplement: Supplementary file 1 [file pharmaceutics-15-00117-s001.zip › pharmaceutics-2086887-supplementary.pdf]
